# Integrative analysis reveals therapeutic potential of pyrvinium pamoate in Merkel cell carcinoma

**DOI:** 10.1172/JCI177724

**Published:** 2025-02-11

**Authors:** Jiawen Yang, James T. Lim, Paul Victor Santiago Raj, Marcelo G. Corona, Chen Chen, Hunain Khawaja, Qiong Pan, Gillian D. Paine-Murrieta, Rick G. Schnellmann, Denise J. Roe, Prafulla C. Gokhale, James A. DeCaprio, Megha Padi

**Affiliations:** 1 University of Arizona Cancer Center, Tucson, Arizona, USA.; 2 Department of Molecular and Cellular Biology, University of Arizona, Tucson, Arizona, USA.; 3 Department of Pharmacology and Toxicology, The University of Arizona R. Ken Coit College of Pharmacy, Skaggs Pharmaceutical Sciences Center, Tucson, Arizona, USA.; 4 Department of Epidemiology and Biostatistics, University of Arizona Mel and Enid Zuckerman College of Public Health, Tucson, AZ, USA.; 5 The University of Arizona College of Medicine, Tucson, Arizona, USA.; 6 The University of Arizona, BIO5 Institute, Tucson, Arizona, USA.; 7 Southern Arizona VA Health Care System, Tucson, Arizona, USA.; 8 Department of Medical Oncology, Dana-Farber Cancer Institute, Boston, Massachusetts, USA.; 9 Department of Medicine, Brigham and Women’s Hospital, Harvard Medical School, Boston, Massachusetts, USA.

**Keywords:** Dermatology, Oncology, Virology, Bioinformatics, Drug therapy, Skin cancer

## Abstract

Merkel Cell Carcinoma (MCC) is an aggressive neuroendocrine cutaneous malignancy arising from either ultraviolet-induced mutagenesis or Merkel cell polyomavirus (MCPyV) integration. Despite extensive research, our understanding of the molecular mechanisms driving the transition from normal cells to MCC remains limited. To address this knowledge gap, we assessed the impact of inducible MCPyV T antigens on normal human fibroblasts by performing RNA-seq. Our data uncovered changes in expression and regulation of Wnt signaling pathway members. Building on this observation, we bioinformatically evaluated various Wnt pathway perturbagens for their ability to reverse the MCC gene expression signature and identified pyrvinium pamoate, an FDA-approved anthelminthic drug known for its antitumor activity in other cancers. Leveraging transcriptomic, network, and molecular analyses, we found that pyrvinium targets multiple MCC vulnerabilities. Pyrvinium not only reverses the neuroendocrine features of MCC by modulating canonical and noncanonical Wnt signaling but also inhibits cancer cell growth by activating p53-mediated apoptosis, disrupting mitochondrial function, and inducing endoplasmic reticulum stress. Finally, we demonstrated that pyrvinium reduces tumor growth in an MCC mouse xenograft model. These findings offer a deeper understanding of the role of Wnt signaling in MCC and highlight the utility of pyrvinium as a potential treatment for MCC.

## Abstract 2

By integrating network and molecular analyses, we revealed that the Wnt signaling dynamics in response to MCPyV plays an important role in MCC development, and identified the therapeutic potential of pyrvinium pamoate inhibiting MCC through multiple pathways.

## Introduction

Merkel cell carcinoma (MCC) is a rare yet highly aggressive neuroendocrine skin cancer, displaying variable incidence rates of approximately 0.3–1.6 per million people across different geographic regions. With metastasis occurring in more than a third of patients with MCC, the disease’s morbidity rate is alarmingly high, resulting in an estimated 5-year overall survival rate of 51% for local, 35% for nodal involvement, and 14% for metastatic MCC (1–5). The current standard of care for MCC involves surgical intervention followed by adjuvant radiation therapy to target the primary tumor or the draining lymph node basin (1). Historically, chemotherapeutic regimens employing combinations of platinum and drugs like etoposide, taxanes, and anthracyclines have been utilized for metastatic MCC cases not amenable to surgery. However, the response rates to chemotherapy in metastatic MCC ranged from 20%–61%, and progression-free survival after chemotherapy was disappointingly limited (1, 6). In 2016, immunotherapy marked a significant advancement in MCC treatment with the introduction of PD1/PDL1 immune-checkpoint inhibitors (ICI), showing efficacy in some patients with MCC. Nevertheless, the responsiveness to ICI therapy remains limited to approximately 50% of patients and many responding patients become resistant to continued therapy (7, 8). Hence, the clinical landscape for MCC still lacks effective and broadly applicable therapeutic agents. Addressing this knowledge gap and identifying alternative treatment options with improved efficacy is of the utmost importance in the pursuit of better outcomes for patients with MCC.

The search for effective drugs targeting vulnerabilities in MCC requires a comprehensive understanding of its development. MCC can arise from either Merkel cell polyomavirus (MCPyV) infection or UV exposure or both, with MCPyV being present in approximately 80% of MCC (9, 10). MCPyV-positive (MCCP) and MCPyV-negative tumors (MCCN) exhibit distinct genetic profiles. MCCN is characterized by a high tumor mutational burden (TMB) with recurrent mutations in *TP53* and *RB1*, whereas MCCP shows a low TMB and lacks hallmark mutations (11–16). Both subtypes share common overexpressed surface markers associated with normal Merkel cells and neuroendocrine tumors (NET) (17–19). Merkel cells are not thought to be the cell of origin of MCC; instead, B cells, dermal fibroblasts, keratinocytes, and neural progenitors have all been proposed as candidates, with dermal fibroblasts being the only cell type demonstrated to support MCPyV viral replication (17). Based on previous studies focusing on the function of the 2 MCPyV antigens, investigators have identified multiple targeted therapies, including but not limited to: activating WT p53 (9, 10), specifically in the context of MCCP (18); inhibiting LSD1, as the complex formed by MCPyV small T antigen with MYCL and EP400 activates the expression of LSD1 (19, 20); inhibiting survivin (21, 22), which is derepressed due to the sequestration of RB1 by large T antigen; and inhibiting EZH2, a histone-lysine N-methyltransferase linked to tumorigenesis via epigenetic silencing of tumor suppressor genes (23–25). However, these drugs have not yet been approved for MCC patients and there is still a need for more effective targeted therapies in this aggressive cancer type.

The Wnt signaling pathway is an intricate network of protein interactions, primarily associated with embryonic development, cell morphogenesis, and proliferation (26, 27). Canonical Wnt signaling and nuclear β-catenin activation are well-known to be associated with tumorigenesis, especially in the context of colon cancer and melanomas (28, 29). Noncanonical Wnt signaling is known to induce terminal neuron differentiation (30, 31). One of its ligands, WNT5A, is notably associated with increased cell motility and invasion, and its expression correlates with higher tumor grade in melanoma (32). In MCC, on the other hand, although previous genomic studies have revealed alterations in Wnt pathway members (33, 34), β-catenin remains localized to the membrane rather than the nucleus, and WNT5A is not expressed (35–37). The functional role of Wnt signaling in MCC therefore remains unclear.

In this study, we perform a wide range of bioinformatic and experimental analyses to identify members of the canonical and noncanonical Wnt signaling pathways involved in MCC development. We then characterize the effect of pyrvinium pamoate, an FDA-approved Wnt inhibitor that has shown antitumor potential in other cancer types, including pancreatic cancer (38, 39), colorectal cancer (40–44), breast cancer (45, 46), acute myeloid leukemia (46–48), and glioblastoma (48, 49). The multifaceted mechanism of action (MOA) of pyrvinium includes, but is not limited to, inhibiting the canonical Wnt pathway (39, 40, 42–44), disrupting mitochondrial function (50, 51), activating the unfolded protein response (UPR) (50, 52), inhibiting tumor stemness (46–48) and impeding PD1/PDL-1 interaction (53). Here, we demonstrate that MCC is sensitive to pyrvinium and explore the impact of pyrvinium’s diverse range of MOAs on the tumorigenic features of MCC.

## Results

### Wnt signaling genes are perturbed by MCPyV and in MCC tumors.

**Figure 1:**
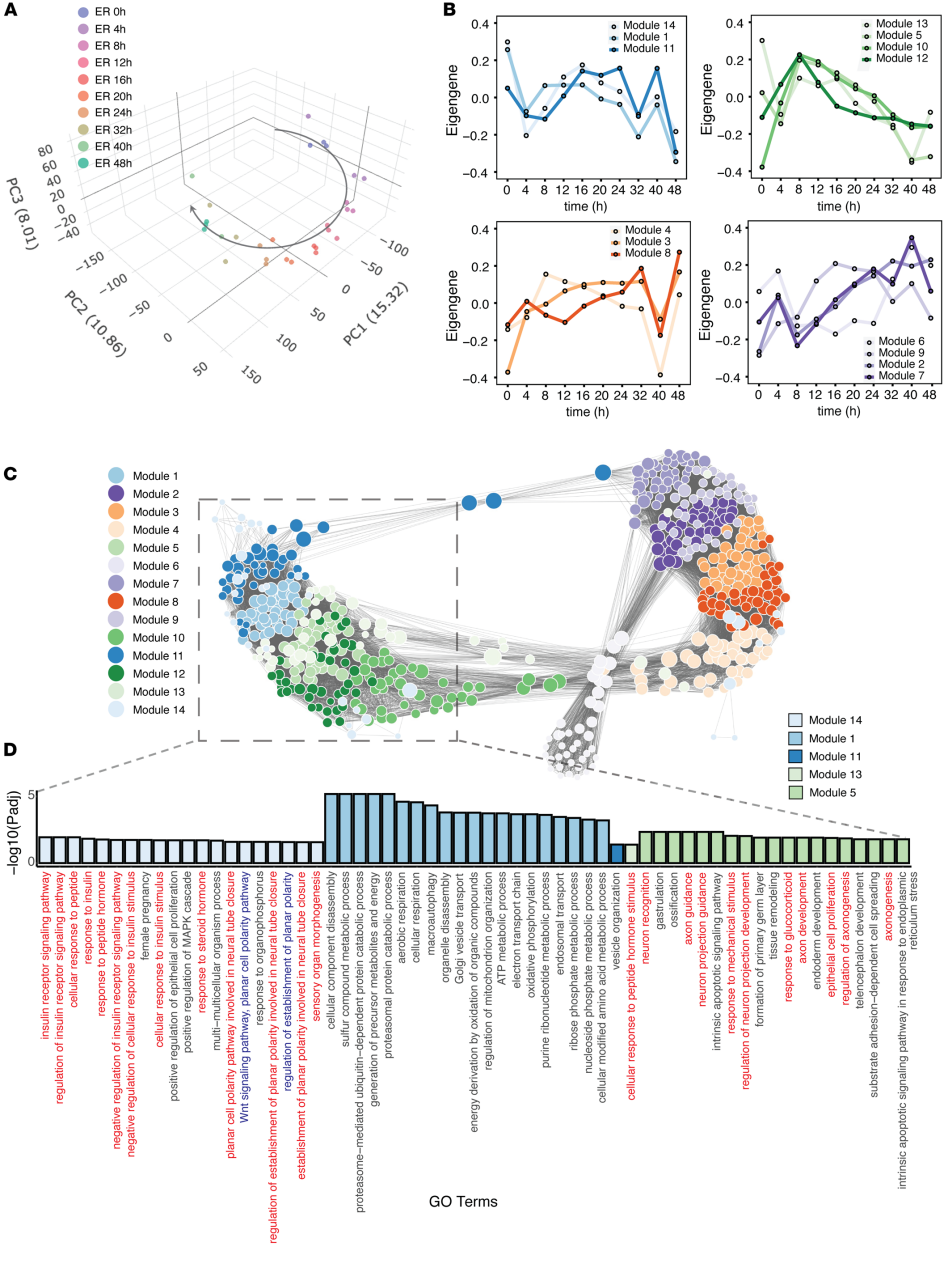
MCPyV-perturbed cell model reveals signaling pathways altered during MCC development. IMR90 normal human fibroblasts expressing inducible MCPyV early region (ER) were subjected to bulk RNA-seq. (**A**) Principal component analysis (PCA) performed on all 13,870 expressed genes in the time series RNA-seq data. (**B**) The eigengenes of the 14 WGCNA modules were projected onto each time point and the modules were grouped by their dynamic patterns using hierarchical clustering. (**C**) Force-directed network of hub genes in the 14 WGCNA modules. The attraction forces between nodes were defined by the topological overlap matrix and were inversely proportional to the length of edges in the graph. (**D**) GO term enrichment analysis of each WGCNA gene module. The terms are ranked by adjusted *P* value, and the top-ranked terms are shown. Neuroendocrine related terms are highlighted in red, Wnt signaling related terms are highlighted in blue.

In an effort to gain deeper insight into the dynamic regulatory events driving MCC tumorigenesis, we established a time-series cell line model using IMR90 normal human embryonic lung fibroblasts. These cells were transduced with a lentivirus containing the MCPyV L21 early region, which codes for both the small (ST) and truncated large T (LT) antigens, or GFP (as a control), under the control of a doxycycline-inducible promoter. To characterize the host transcriptional response to MCPyV-ER, we performed RNA-seq in triplicate at 10 time points from 0 to 48 hours (Supplemental Figure 1A and Supplemental Spreadsheet 1; supplemental material available online with this article; https://doi.org/10.1172/JCI177724DS1). The raw reads were aligned to a concatenated human and viral genome to confirm that ST and LT were expressed in IMR90-ER samples in a time-dependent manner (Supplemental Table 1). Principal component analysis (PCA) revealed a trajectory representing a dynamic host transcriptome change induced by MCPyV-ER (Figure 1A). We then examined the genes that were differentially expressed between the early region and GFP samples at 48 hours (Supplemental Spreadsheet 2). As expected, the most significantly enriched GO terms were related to DNA replication and cell cycle, consistent with LT binding and inactivating Rb. In addition, we found enrichment for a wide range of other pathways (Supplemental Spreadsheet 3), including “regulation of neurogenesis” (GO:0050767; *P*_adj_ = 2.01 × 10^–2^; *OR* = 1.22) and “axonogenesis” (GO:0007409; *P*_adj_ = 8.81 × 10^–4^; *OR* = 1.28). Examining the genes annotated to these neuronal GO terms, we found that some MCC marker genes, including *ENO2*, *NEFM*, *NEFH*, and *HES6*, were increased by MCPyV-ER (Supplemental Figure 1, B-E; Supplemental Spreadsheet 2). However, we did not observe changes in the MCC markers *CHGA*, *ATOH1*, *SOX2*, and *INSM1*, likely due to cell-type–specific gene regulation in IMR90 cells.

To identify functional modules of host genes perturbed by the early region, we conducted Weighted Gene Coexpression Network Analysis (WGCNA) on the IMR90-ER data across all time points. This analysis led to the identification of 14 modules, which we further characterized based on their dynamic patterns (Figure 1B) and module eigengenes (MEs) (Supplemental Figure 2A). We visualized the top hub genes within each module and the interrelationships between the modules using a force-directed layout that places 2 genes closer together if they have a stronger correlation (Figure 1C). To gain insight into the biological functions associated with each module, we performed Gene Ontology (GO) enrichment analysis. The network visually separated into 2 components; the blue (Module 1, 11, and 14) and green (Module 5, 10, 12, and 13) modules clustered together on the left side, whereas the purple and orange modules clustered together on the right side and were enriched in cell cycle and metabolism pathways (Supplemental Figure 3). Among the blue modules, we observed that Module 14 genes were strongly enriched for the Wnt signaling pathway and steroid hormone response, Module 1 was enriched for cellular respiration, and Module 11 was enriched for vesicle organization, whereas the green modules were enriched for neuronal pathways (e.g. “axon development” and “neuron projection guidance” in Module 5; Figure 1D). The eigengene analysis (Figure 1B) and module-trait (with time being considered the trait) relationship analysis (Supplemental Figure 2B) indicated that the gene expression signatures of the blue and green modules were initially distinct from each other but gradually converged over time.

**Figure 2:**
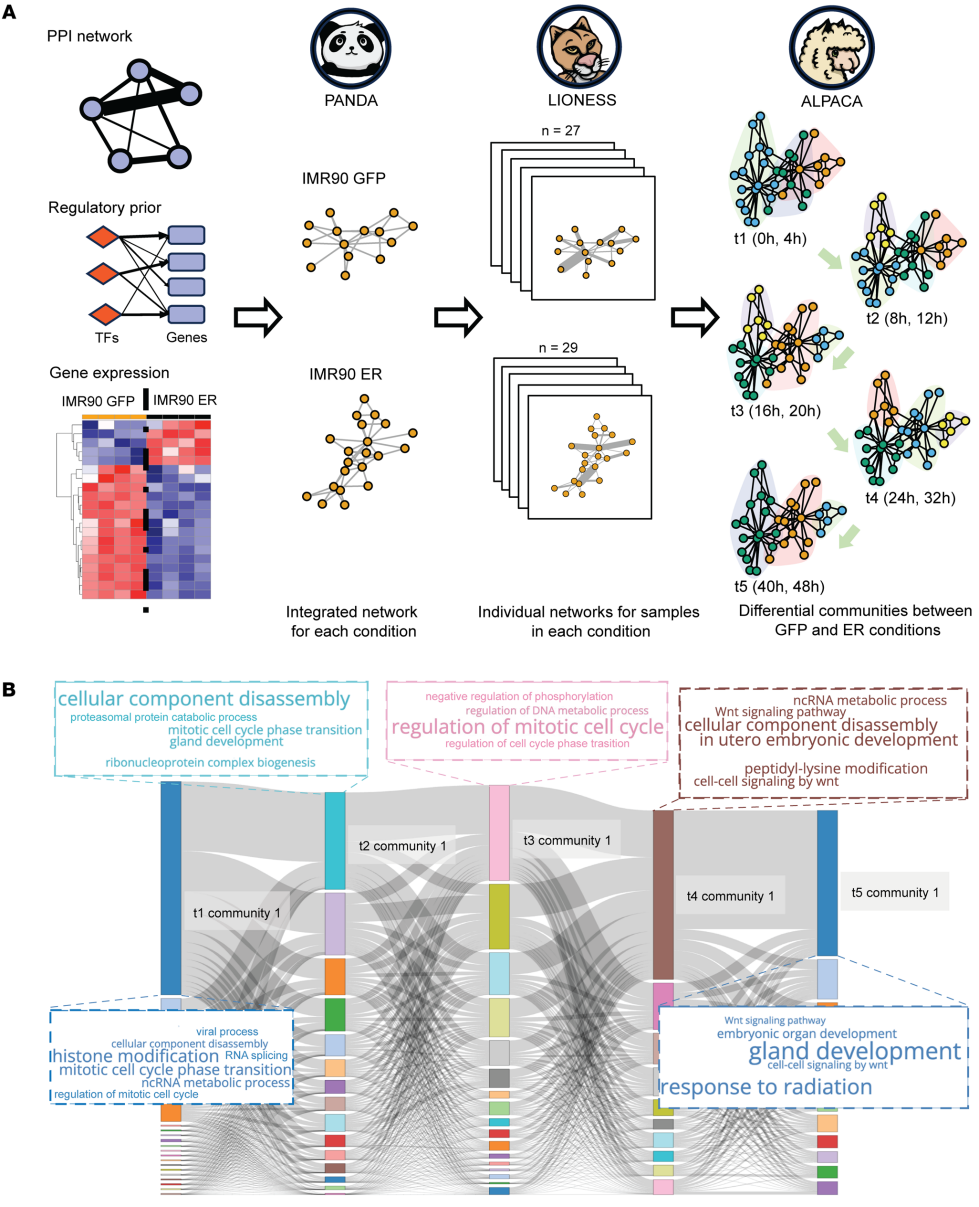
Identifying areas of active gene regulation in IMR90 cells expressing MCPyV T antigens. (**A**) Graphic workflow of regulatory network analysis. RNA-seq data was integrated with TF motif binding prior and TF protein-protein interactions to infer sample-specific regulatory networks using PANDA and LIONESS. IMR90-ER networks were grouped into 5 time periods and compared with IMR90-GFP networks from the same time period using ALPACA, to identify differential modules. (**B**) Sankey plot shows the dynamics of differential network communities detected by the workflow shown in **A**. Each vertical bar represents a differential community, with the size of the bar proportional to the number of genes in the community. Ribbons between adjacent bars represent the number of overlapping genes. Word cloud in the same color as the gene module annotates the enriched biological functions of genes inside the module (font size reflects the *P*_adj_ value).

To determine how host genes were being transcriptionally regulated in the presence of the early region, we inferred the transcriptional regulatory networks active in both IMR90-GFP and IMR90-ER cells by integrating RNA-seq data, protein-protein interactions (PPI) and transcription factor–motif (TF-motif) information using PANDA (54) and LIONESS (55) in 5 distinct time periods between 0 to 48 hours. We then employed ALPACA (56) to identify differentially regulated gene sets (or “communities”) that best distinguish the IMR90-ER and IMR90-GFP networks during each of these 5 time periods (Figure 2A). Alongside capturing general biological processes like cell cycle, nucleic acid synthesis, protein synthesis, metabolic pathways, and histone modification, which are typically regulated during the transformation process, we also found that ALPACA community 1, the largest differential gene community between IMR90-ER and IMR90-GFP at later time periods, was enriched in Wnt signaling (*P*_adj_ = 8.79 × 10^–5^, *OR* = 1.70) and embryonic development (*P*_adj_ = 7.10 × 10^–5^, *OR* = 1.74) (Figure 2B) (Supplemental Spreadsheet 4, sheet: “er_versus_gfp_t5_module_1”).

**Figure 3:**
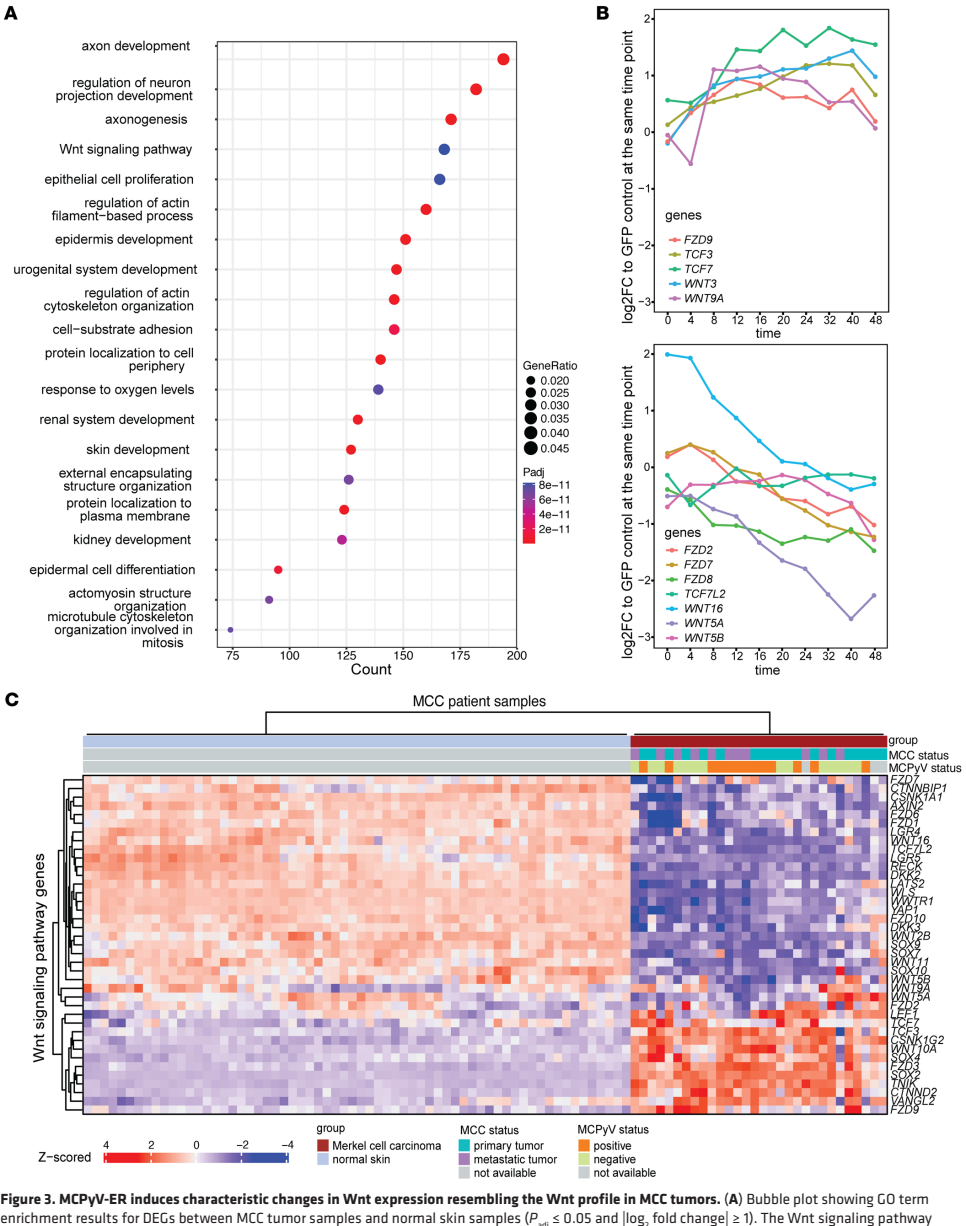
MCPyV-ER induces characteristic changes in Wnt expression resembling the Wnt profile in MCC tumors. (**A**) Bubble plot showing GO term enrichment results for DEGs between MCC tumor samples and normal skin samples (*P*_adj_ ≤ 0.05 and |log_2_ fold change| ≥ 1). The Wnt signaling pathway ranked as one of the most significantly enriched pathways. (**B**) Log_2_ fold change of selected Wnt gene expression levels in IMR90-ER samples, relative to the IMR90-GFP samples at the corresponding time points. The genes were categorized into 2 sets based on their expression dynamics. (**C**) Heatmap of Wnt signaling pathway genes in MCC tumor samples and normal skin samples. Two distinct trends in Wnt gene expression were observed, with several genes, including *FZD7*, *WNT16*, *TCF3*, and *TCF7*, showing trends consistent with those observed in our IMR90 model.

The IMR90 fibroblast cell model is incomplete and does not fully reflect the true cell of origin of MCC, which remains unknown. We therefore compared our analysis of the IMR90 cell lines to transcriptomic data from MCC patient samples. We focused on genes that are significantly differentially expressed (*P*_adj__j_ ≤ 0.05, |log2 fold change| ≥ 1) between MCC tumors versus normal skin samples in a previously published dataset (GSE39612). Among these tumor DEGs, the strongest enrichment was associated with the Wnt signaling pathway (*P*_adj_ = 8.24 × 10^–11^, *OR* = 1.67) and neural development pathways including axon development (*P*_adj_ = 9.81 × 10^–17^, *OR* = 2.49), regulation of neuron projection development (*P*_adj_ = 5.12 × 10^–15^, *OR* = 2.42), and axonogenesis (*P*_adj_ = 3.51 × 10^–14^, *OR* = 2.41) (Figure 3A). In a WGCNA analysis of samples from patients with MCC (Supplemental Figure 4, A and B), we observed that Wnt pathway genes exhibited close coexpression with genes enriched for keratinocyte and skin development pathways (Supplemental Figure 4C).

Next, we examined the temporal pattern of Wnt gene expression in more detail. In the IMR90 cell line model, we observed that canonical Wnt genes, such as *WNT3*, *TCF7*, and *TCF3*, were upregulated over time in the early region samples relative to GFP control, whereas noncanonical Wnt genes, such as *WNT5A*, *WNT5B*, and *WNT16*, were downregulated over time (Figure 3B and Supplemental Figure 5A). To validate this pattern of expression, we transduced normal human dermal fibroblasts (nHDFs) with the lentivirus containing the MCPyV L21 early region or vector control under a dox-inducible promoter and performed RNA-seq at 48 and 96 hours after induction. Normal human dermal fibroblasts can support productive MCPyV infection and thus represent a more likely cell of origin for MCC than IMR90s (17). In the nHDF-ER cells, we again found significant enrichment for neuronal GO terms at 48 hours (e.g., GO:0010975, regulation of neuron projection development; *P*_adj_ = 1.88 × 10^–5^, *OR* = 1.63) and upregulation of selected MCC marker genes including *NOTCH3*, *EZH2*, *HES6*, *HES1*, and *NEFH* (Supplemental Spreadsheets 5 and 6, and Supplemental Figure 6A)**.** All the Wnt signaling genes that were perturbed in IMR90-ER cells, except for *TCF7*, exhibited similar trends in nHDF-ER cells, particularly the noncanonical Wnt signaling genes *WNT5A*, *WNT5B*, and *WNT16*, and the canonical Wnt signaling genes *WNT3* and *TCF3* (Supplemental Figure 6B). We confirmed these trends for *TCF3* and *WNT5A/B* at the protein level (Supplemental Figure 6C).

Finally, we looked more closely at the correlation between the expression of Wnt genes and a curated set of neuroendocrine-related (NE-related) genes that were upregulated in IMR90 cells during the previously defined 5 time periods. IMR90-ER cells demonstrated an increased absolute Pearson correlation coefficient between the 2 sets of genes, whereas IMR90-GFP controls did not show any change in correlation, indicating that the Wnt signaling pathway is coregulated with MCC and NE markers specifically in the presence of the early region. In particular, canonical Wnt genes such as *WNT3*, *TCF7*, and *TCF3* were upregulated over time and positively correlated with NE or MCC markers, whereas noncanonical Wnt genes like *WNT5A* and *WNT16* showed strong negative correlation with MCC markers (Supplemental Figure 5B). The same pattern of upregulation of canonical Wnt genes and downregulation of noncanonical Wnts was found in the MCC tumor dataset (Figure 3C). Therefore, we hypothesized that MCC development and neuroendocrine differentiation requires suppression of noncanonical Wnt signaling and activation of canonical Wnt signaling. Targeting the Wnt pathway could potentially reverse the neuroendocrine features of MCC.

### Pyrvinium pamoate effectively reverses the MCC signature compared with other Wnt perturbagens.

**Figure 4:**
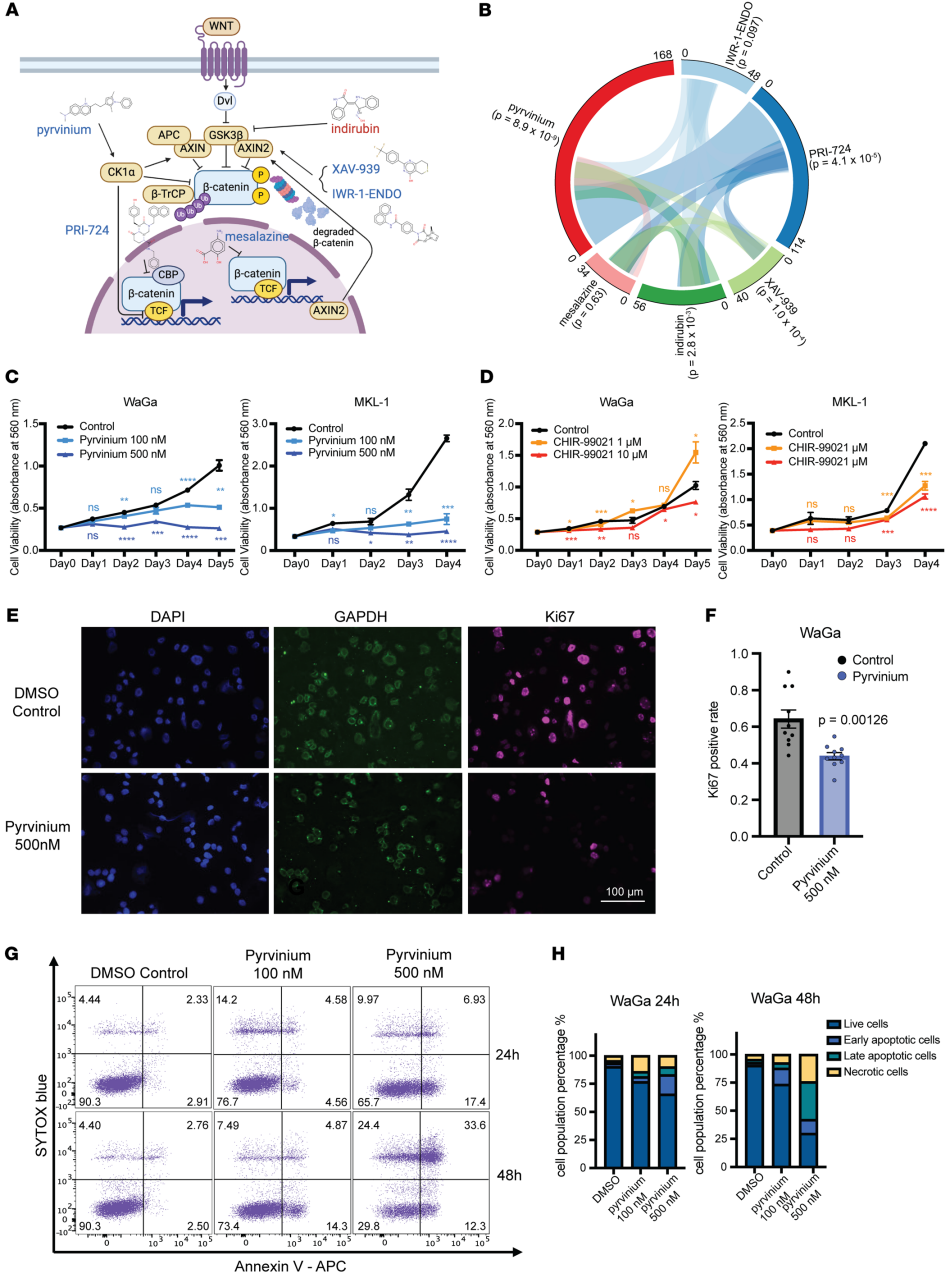
Characterization of pyrvinium pamoate as an effective perturbagen against MCC. (**A**) Simplified diagram of commercially available Wnt signaling perturbagens in LINCS L1000 dataset and their reported MOAs. (Created in BioRender. Yang, J. (2025) https://BioRender.com/v23e056) (**B**) Circos plot showing pairwise comparison between MCC signature genes (MCC1000) and top drug-perturbed genes (ranked by *z*-scored log_2_[fold change]) under different drug treatments. Statistical significance was determined by Fisher’s exact test. (**C** and **D**) Cell proliferation assay in WaGa and MKL-1 cells, under treatment with pyrvinium, CHIR-99021, and DMSO control. Statistical significance was determined by unpaired 2-sample, 2-tailed *t* test (****P < 0.0001; ***P < 0.001; **P < 0.01; *P < 0.05). (**E**) Representative immunofluorescence (IF) images of WaGa cells treated with 500 nM pyrvinium and DMSO, with Ki67 staining as a proliferation marker, GAPDH as internal control, and DAPI for nucleus. Scale bar: 100 μm. (**F**) Bar graph showing the quantification of the Ki67-positive cell count relative to the total nucleus count in IF images of WaGa cells treated with 500 nM pyrvinium or DMSO. Data are presented as mean ± SEM (*n* = 10, *P* = 0.00126). Statistical significance was determined by unpaired 2-sample, 2-tailed *t* test. (**G**) Flow cytometry analysis showing the levels of Annexin V-APC and SYTOX blue staining to assess apoptotic populations in WaGa cells treated with varying doses and durations. (**H**) Bar graphs showing the quantification of different populations in pyrvinium-treated WaGa cells at 24 hours and 48 hours separately.

We next set out to identify small-molecule perturbagens that could target the Wnt signaling pathway and reverse the gene expression changes observed in MCC. To do this, we leveraged the LINCS L1000 data to examine the impact of various Wnt signaling perturbagens on reversing the gene expression signature of MCC (Figure 4A). We focused on the drugs pyrvinium pamoate, XAV-939, IWR-1-ENDO, mesalazine, PRI-724, and indirubin, as these were the only compounds annotated by LINCS to perturb the Wnt pathway that were also commercially available. Utilizing the samples from patients with MCC in the GSE39612 dataset, we generated a set of MCC signature genes by computing the average Pearson correlation coefficient between the expression of each gene with a set of known MCC marker genes: *ENO2*, *NEFM*, *NEFH*, *NMB*, *HES6*, *SOX2*, *ATOH1*, and *CHGA*. We selected the top 500 genes with the highest and lowest correlation scores, which we call the MCC1000 signature (Supplemental Spreadsheet 7). By comparing the MCC1000 with the top 1000 differentially expressed genes from each drug perturbation, we discovered that pyrvinium (*P*_adj_ = 8.9 × 10^–9^, *OR* = 1.97), a CK1α activator, which promotes the phosphorylation and degradation of β-catenin by proteosomes (44), exhibited the highest efficiency in reversing MCC1000 expression when compared with other Wnt perturbagens (Figure 4B and Supplemental Figure 7, A–F). To validate our in-silico findings, we treated MCC cell lines with pyrvinium pamoate. Pyrvinium effectively inhibited the growth of MCC cell lines (Figure 4C), even outperforming the previously reported effective drug CHIR99021, a GSK3β inhibitor sharing the same MOA as indirubin in the L1000 dataset (Figure 4D). The MTT cell growth assay demonstrated that pyrvinium significantly hindered MCC growth at concentrations as low as 100 nM. Immunofluorescence revealed that pyrvinium significantly reduced the expression of Ki67, a nuclear proliferation marker (Figure 4, E and F). Furthermore, flow cytometry analysis showed that pyrvinium can induce cell apoptosis in a dose- and time-dependent manner in 4 different MCC cell lines (Figure 4, G and H, and Supplemental Figure 8).

### Pyrvinium pamoate reveals the role of Wnt signaling in maintaining a neuroendocrine state.

**Figure 5:**
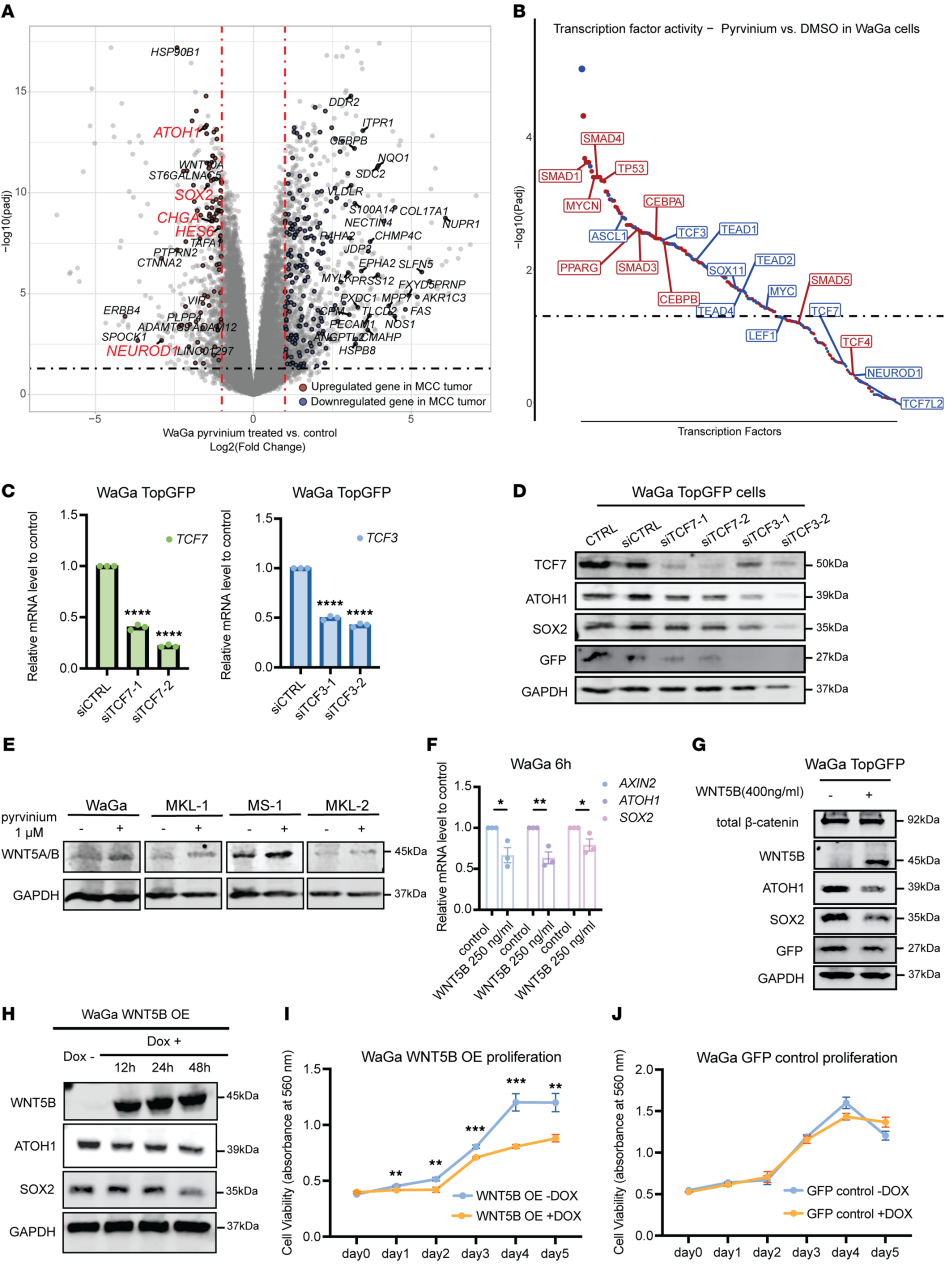
Pyrvinium pamoate reverses neuroendocrine and Wnt signaling signature in MCC cells. (**A**) Volcano plot of DEGs in WaGa cells treated with pyrvinium compared with DMSO for 24 hours. DEGs with *P*_adj_ ≤ 0.05 and |log_2_ fold change|≥ 1 that show a reversed expression trend relative to MCC versus normal skin are highlighted in red (upregulated in MCC) or blue (downregulated in MCC). Known MCC marker genes are labeled in red text. (**B**) Scatter plot of predicted master regulator activity levels in pyrvinium-treated MCC versus DMSO-treated MCC cells. Blue (or red) indicates regulators with decreased (or increased) activity. (**C**) Relative mRNA levels of *TCF7* and *TCF3* in WaGa TopGFP cells treated with siRNA control, siTCF7, and siTCF3, as measured by RT-qPCR. Statistical significance determined by unpaired 2-sample *t*-test. (**D**) Protein levels of ATOH1, SOX2, and GFP, in untreated WaGa TopGFP cells and the cells treated with siRNA negative control, siTCF7, and siTCF3. (**E**) Protein levels of WNT5A/B following pyrvinium treatment for 24 hours in MCC cell lines. (**F**) Relative mRNA levels of *AXIN2*, *ATOH1*, and *SOX2* in WaGa cells treated with recombinant hWNT5B protein for 6 hours, as measured by RT-qPCR. Statistical significance determined by unpaired 2-sample, 2-tailed *t* test. (**G**) Protein levels of total β-catenin, WNT5B, ATOH1, SOX2, and GFP following 6 hours of treatment with recombinant hWNT5B in WaGa TopGFP cells. (**H**) WNT5B, ATOH1, and SOX2 protein levels at 0, 12, 24, and 48 hours after 1 μg/mL doxycycline induction in WaGa WNT5B OE cells. (**I** and **J**) Cell viability in WaGa WNT5B OE cells and GFP control cells with or without 1 μg/mL doxycycline. Statistical significance between dox+ and dox– conditions on the same day was assessed by unpaired 2-sample, 2-tailed *t* test. (****P < 0.0001; ***P < 0.001; **P < 0.01; *P < 0.05).

According to multiple studies on pyrvinium’s potential as an antitumor agent, its main mechanisms of action (MOAs) include the inhibition of canonical Wnt signaling, mitochondrial inhibition, and the activation of unfolded protein response (38, 44, 50, 57). To gain a comprehensive understanding of pyrvinium’s effect on MCC, we conducted RNA-seq on WaGa and MKL-1 cells treated with 1 μM of pyrvinium, using DMSO as vehicle control, for 6 hours and 24 hours (*n* = 3). In pyrvinium-treated MCC cells, we observed downregulation of MCC marker genes, such as *ATOH1*, *SOX2*, *CHGA*, *HES6*, and *NEUROD1* (Figure 5A). To identify potential mechanisms by which pyrvinium could reverse MCC marker gene expression, we set out to predict master regulator activity in pyrvinium-treated MCC cells using the state-of-the-art TF activity prediction algorithm, VIPER, in conjunction with the human TF regulon database-DoRothEA. Upon pyrvinium treatment, the predicted protein activity of several MCC master regulators, including p53, MYCN, SMADs, and SOX11, exhibited an opposite trend to that previously reported during MCC development (Figure 5B) (9, 20, 58–61). DoRothEA is a generic TF regulon database that is agnostic to cell type. We therefore repeated the VIPER analysis using MCCP-specific regulons built using ARACNe. This analysis revealed additional MCC-specific regulators such as POU4F3, HES6, and ATOH1 whose activities were reversed by pyrvinium treatment (Supplemental Figure 9, A and B).

Consistent with pyrvinium’s reported activity as a canonical Wnt inhibitor, we observed that TCF3, the main effector of canonical Wnt signaling, had higher expression in IMR90-ER and nHDF-ER cells, higher predicted activity in IMR90-ER cells, and lower predicted activity after pyrvinium treatment in MCC cell lines. Although previous studies showed low nuclear β-catenin expression in MCC (37), TCFs can be activated independently of nuclear β-catenin (62). We therefore hypothesized that MCC development involves direct activation of TCF family members. We introduced siRNAs targeting *TCF3* and *TCF7* into WaGa cells transduced with a TopGFP reporter. The TopGFP plasmid expresses GFP under the control of a 7x TCF/LEF promoter cassette, reflecting canonical Wnt signaling activity levels. After introducing the siRNAs into WaGa TopGFP cells, we confirmed that TCF/LEF activity was significantly reduced (Figure 5C). We then found that knocking down *TCF3*, and, to a lesser extent *TCF7*, reduced the protein levels of neuroendocrine markers ATOH1 and SOX2 (Figure 5D; please see Supplemental Spreadsheet 8 for statistics and quantification of all Western blots). This confirms that MCC cells require TCFs to maintain neuroendocrine marker expression.

In pyrvinium-treated MCC cells, we observed not only the expected inhibition of canonical Wnt signaling (Figure 5B and Supplemental Figure 9C) but also a significant mRNA upregulation of *WNT5A* and *WNT5B*, which are Wnt ligands known to activate the noncanonical Wnt signaling pathway, which we confirmed with RT-qPCR analysis (Supplemental Figure 9, D and E). Consistent with this, Western blot results showed a modest increase in WNT5A/B levels in WaGa, MKL-1, and MS-1 cells treated with 1 μM of pyrvinium (Figure 5E). WNT5A has been previously reported to promote neuron differentiation and morphological development (30, 31) and to be highly repressed in MCC tumors (35). We therefore asked whether perturbation of noncanonical Wnt alone could affect the neuroendocrine and Wnt signatures seen in MCC. Treating WaGa cells with WNT5B human recombinant protein resulted in decreased transcription of master neural development regulators, *ATOH1* and *SOX2*, as well as reduced expression of the canonical Wnt target gene *AXIN2* (Figure 5F), as evident in our RT-qPCR results. Furthermore, our Western blot results showed a modest decrease in ATOH1, SOX2, and GFP levels upon WNT5B recombinant protein treatment (Figure 5G and Supplemental Figure 9F). However, β-catenin levels remained unchanged following WNT5B recombinant protein treatment. This suggests that WNT5B inhibits MCC regulators and canonical Wnt activity through a β-catenin–independent mechanism. To confirm the effect of WNT5B on MCC, we established a doxycycline-inducible WNT5B overexpression (WNT5B OE) WaGa cell line. Overexpression of WNT5B resulted in decreased protein levels of ATOH1 and SOX2, consistent with the recombinant protein treatment results (Figure 5H). A cell growth assay using WaGa WNT5B OE and WaGa GFP control cells suggested that the decrease in ATOH1 and SOX2 protein levels following WNT5B overexpression may be accompanied by inhibitory effects on MCC cell growth (Figure 5, I and J). In summary, the neuroendocrine features of MCC tumors can be inhibited by downregulating canonical Wnt or upregulating noncanonical Wnt, both of which can be enabled by pyrvinium. This is one mechanism by which pyrvinium may reverse the process of MCC carcinogenesis.

### Transcriptome analyses reveal other mechanisms of action of pyrvinium.

**Figure 6:**
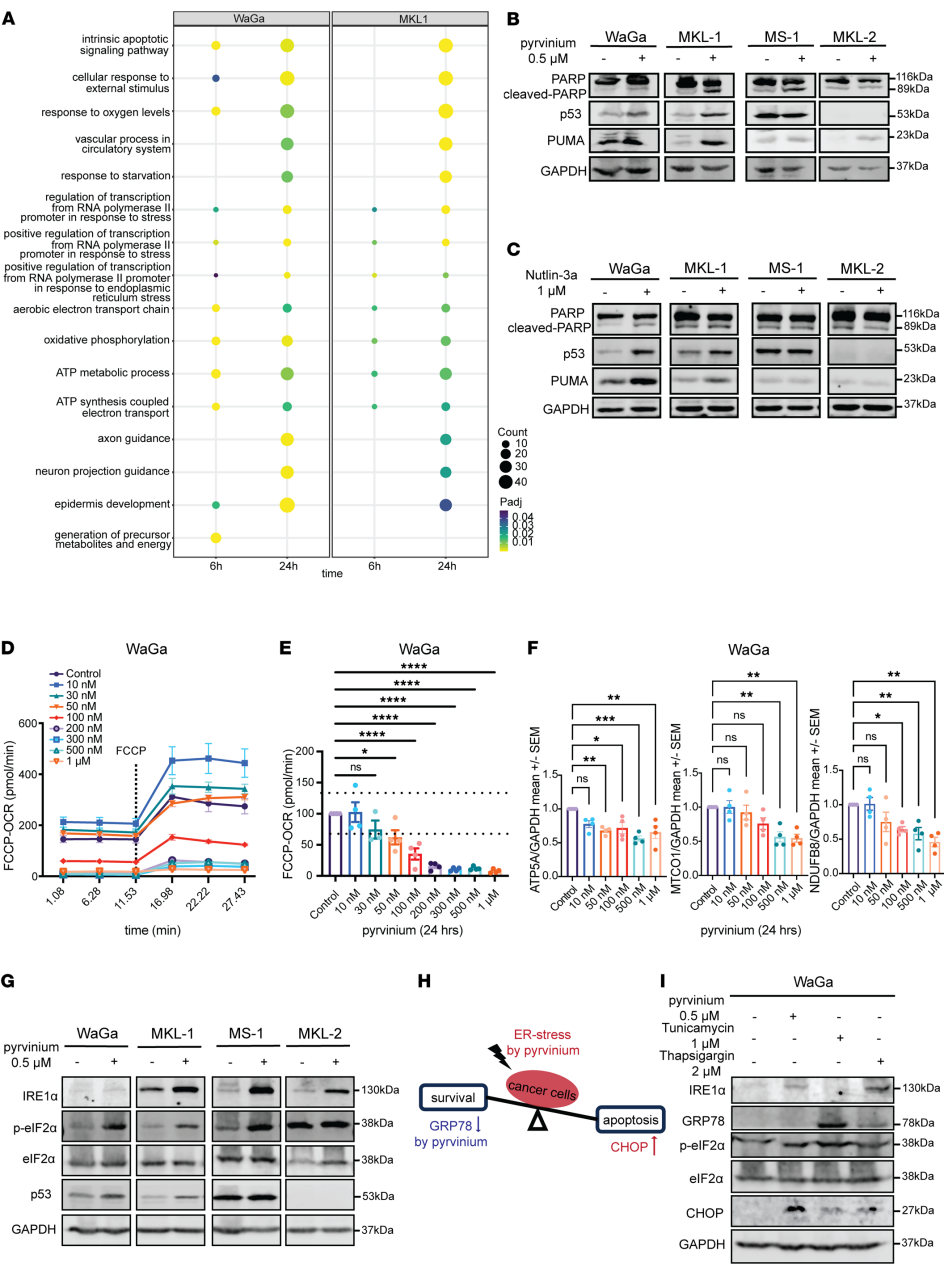
Pyrvinium targets multiple vulnerabilities of MCC. (**A**) GO term enrichment for 1 μM pyrvinium-treated WaGa and MKL1 cells at 6 and 24 hours. Size of dot indicates number of genes annotated to the GO term, and color reflects the adjusted *P* value from the hypergeometric test. GO terms are primarily ranked by significance in the 24-hour MKL1 analysis. (**B** and **C**) Protein levels of p53, cleaved-PARP, and PUMA in *TP53* WT cell lines (WaGa, MKL-1) and *TP53*^Mut^/*TP53*^–/–^ cell lines (MS-1, MKL-2) 24 hours after treatment with 0.5 μM pyrvinium and 1 μM Nutlin-3a. (**D**) Representative data displayed as a line chart showing basal respiration level and maximal respiration capacity (after FCCP injection) at each time point (means ± SEM, *n* = 4). (**E**) Seahorse OCR analysis measuring uncoupled OCR in WaGa cells treated with different doses of pyrvinium for 24 hours compared with DMSO treated cells. Statistical significance determined by Kruskal-Wallis H Test followed by Dunnett’s post hoc test. (**F**) Quantification of OXPHOS protein levels from Western blot (Supplemental Figure 12B). All values are presented relative to mean GAPDH expression in vehicle control samples (means ± SEM, *n* = 4 replicate blots). Statistical significance determined by ANOVA followed by Dunnett’s multiple comparison test. (****P < 0.0001; ***P < 0.001; **P < 0.01; *P < 0.05). (**G**) Protein levels of endoplasmic reticulum stress markers in MCC cells treated with pyrvinium for 6 and 24 hours. (**H**) A graphic model illustrating pyrvinium’s effect on the balance between endoplasmic reticulum stress and UPR signaling. (**I**) Proteins levels of Unfolded Protein Response (UPR) markers in WaGa cells after 24 hours of treatment with pyrvinium or other endoplasmic reticulum stress inducers.

To characterize the genome-wide impact of pyrvinium on MCC cells, we performed GO term enrichment on all the differentially expressed genes (DEGs) following pyrvinium treatment. The most enriched GO terms were strongly associated with the “intrinsic apoptotic signaling pathway” (GO: 0097193, MKL1-24h, *P*_adj_ = 4.24 × 10^–5^), “oxidative phosphorylation” (GO: 000619, MKL1-24h: *P*_adj_ = 1.23 × 10^–2^), and “axon guidance” (GO: 0007411, MKL1-24h: *P*_adj_ = 7.98 × 10^–4^), among others (Figure 6A). To further integrate the direction of fold change and their linkages to enriched pathways, we used all the DEGs from 24 hours after pyrvinium treatment, divided them into upregulated (log_2_ fold change > 1) and downregulated (log_2_ fold change < –1) clusters, performed KEGG pathway overrepresentation analysis, and constructed a gene-biological concepts network for each regulation direction cluster (Supplemental Figure 10). This network highlighted the “p53 signaling pathway” (hsa04115, MKL1-up: *P*_adj_ = 1.59 × 10^−6^, WaGa-up: *P*_adj_ = 1.42 × 10^−5^) as the top activated pathway and “oxidative phosphorylation” (hsa00190, MKL1-down: *P*_adj_ = 1.47 × 10^−5^, WaGa-down: *P*_adj_ = 1.19 × 10^−8^) as the top inhibited pathway in both cell lines. Interestingly, the network also highlighted “Small cell lung cancer” (hsa05222, MKL-1-up: *P*_adj_ = 9.7 × 10^−3^) and “human papillomavirus infection” (hsa05165, MKL-1-up: *P*_adj_ = 9.7 × 10^−3^), the biological characteristics of which are similar to MCC. These results suggested that pyrvinium’s effects are specific to neuroendocrine cancer and tumor viruses. Our analysis also revealed strong activation of endoplasmic reticulum stress by pyrvinium treatment in MCC cell lines (Supplemental Figure 11).

### Pyrvinium pamoate induces MCC cell apoptosis through p53-dependent and -independent mechanisms.

p53 functions as an essential tumor suppressor, and loss-of-function mutations in the *TP53* gene are found in approximately 50% of all cancers (63). However, p53 inactivation mutations are less frequently (13%–28%) reported in MCC (11, 12, 64). To determine if the proapoptotic effect of pyrvinium is dependent on WT p53, we used a panel of 4 MCC cell lines: WaGa, MKL-1, MS-1, and MKL-2. WaGa and MKL-1 cell lines are p53 WT cells; MS-1 harbors a *TP53* deletion mutation, resulting in an inactive p53 protein lacking amino acids 251–253; in MKL-2, p53 protein is undetectable due to posttranscriptional repression (65). Our Western blot results showed significant increases in p53, cleaved PARP, and PUMA protein levels in pyrvinium treated WaGa and MKL-1 cells. In contrast, PUMA protein levels did not change in MS-1, but exhibited a modest increase in MKL-2 (Figure 6B). We further compared the effect of pyrvinium in all 4 cell lines with the MDM2 inhibitor Nutlin-3a. Nutlin-3a increased p53, cleaved-PARP, and PUMA protein levels in WaGa and MKL-1 cells, but with lower efficacy than pyrvinium. Nutlin-3a did not increase the levels of cleaved-PARP and PUMA in MS-1 and MKL-2 (Figure 6C). MTT assay results revealed that at the 48-hour time point, the IC_50_ values for pyrvinium in WaGa (0.4217 μM), MKL-1 (0.1104 μM), MS-1 (0.3359 μM), and MKL-2 (0.4362 μM) cells were all in the 100 nanomolar range. In contrast, Nutlin-3a was only effective in WaGa (1.290 μM), and MKL-1 (0.1441 μM) cells and showed no inhibitory effect in MS-1 and MKL-2 even at 10 μM (Supplemental Figure 12A). Our results suggest that, although p53 activation plays a significant role in cell apoptosis during pyrvinium treatment, there are also p53-independent proapoptotic mechanisms that provide pyrvinium with an advantage as a novel therapy for treating *TP53^mut^* or *TP53^–/–^* MCC.

Pyrvinium has been previously identified as a mitochondrial inhibitor and has demonstrated even greater potency in nutrient-deficient conditions (38, 39, 66). To assess the extent of oxidative phosphorylation inhibition by pyrvinium in MCC cells, we conducted an FCCP-OCR analysis using the Seahorse XF96 Analyzer. Employing dose-dependent treatments of pyrvinium over 24 hours, we evaluated the FCCP-OCR response. Remarkably, we observed a significant decrease in FCCP-uncoupled OCR (a measurement of maximal ETC activity), indicative of electron transport chain impairment, starting from around 50-100 nM of pyrvinium (Figure 6, D and E). Additionally, Western blots performed using the same samples as the Seahorse experiment revealed that pyrvinium treatment reduced the protein levels of multiple components in mitochondrial complexes (NDUFB8 subunit of Complex I, MTCO1 subunit of Complex IV, ATP5A subunit of Complex V, *n* = 4) (Figure 6F and Supplemental Figure 12, B–D). The combined results from OCR and Western blotting indicate that pyrvinium impairs mitochondrial function by inhibiting the expression of mitochondrial complex subunits. Moreover, pyrvinium treatment downregulated numerous mitochondrial protein-coding genes (Supplemental Figure 12E), indicating that the decrease in mitochondrial complex proteins could also arise from direct suppression of mitochondrial gene transcription.

A potential downstream effect of mitochondrial dysfunction is endoplasmic reticulum stress. The endoplasmic reticulum is tightly associated with mitochondria by multiple contact sites and forms special domains called mitochondria–endoplasmic reticulum associated membranes (MAMs). Endoplasmic reticulum stress can be triggered by various intra- or extracellular factors, such as glucose starvation, hypoxia, Ca^2+^ depletion and protein misfolding. In our RNA-seq data, we observed significant enrichment and upregulation of endoplasmic reticulum stress signaling, which could lead to cell apoptosis. Western blot analysis demonstrated that pyrvinium treatment elevated the activity of endoplasmic reticulum stress sensors residing in the endoplasmic reticulum membrane, including increased IRE1α protein levels and increased phosphorylation of eIF2α by PERK in all MCC cell lines, regardless of their p53 status (Figure 6G). The master regulator of unfolded protein response (UPR) signaling, GRP78 (BiP), can mitigate endoplasmic reticulum stress by arresting transient transcription, degrading endoplasmic reticulum–associated proteins and inducing endoplasmic reticulum chaperones to support cell survival. However, in cases of severe endoplasmic reticulum stress, apoptosis is triggered by the effector protein CHOP (52). To investigate changes in endoplasmic reticulum stress and UPR levels with varying pyrvinium dosages, we performed Western blot analysis on WaGa cells treated with pyrvinium for 24 hours at different concentrations. The results indicated that pyrvinium increased endoplasmic reticulum stress and reduced GRP78 levels in a dose-dependent manner, while CHOP levels increased promptly when treated with 500 nM of pyrvinium (Supplemental Figure 12F). Overall, these results suggest that pyrvinium treatment elevates endoplasmic reticulum stress levels and impairs the unfolded protein response, leading to an amplification of endoplasmic reticulum stress and induction of cell apoptosis (Figure 6H). Furthermore, we compared the endoplasmic reticulum–stress inducing capacity of pyrvinium with known endoplasmic reticulum inducers, namely tunicamycin (TM), an N-glycans blocker that induces unfolded protein generation, and thapsigargin (TG), an inhibitor of sarco-endoplasmic reticulum Ca^2+^ ATPase (SERCA) that prevents Ca^2+^ flow from the cytoplasm into the endoplasmic reticulum. We found that 500 nM pyrvinium induced CHOP in MCC cells to a stronger extent than thapsigargin and tunicamycin applied at higher concentrations (Figure 6I). In conclusion, pyrvinium efficiently enhances endoplasmic reticulum stress in all MCC cells, potentially contributing to cell death.

### Pyrvinium pamoate effectively inhibits tumor growth in xenograft mouse model.

**Figure 7:**
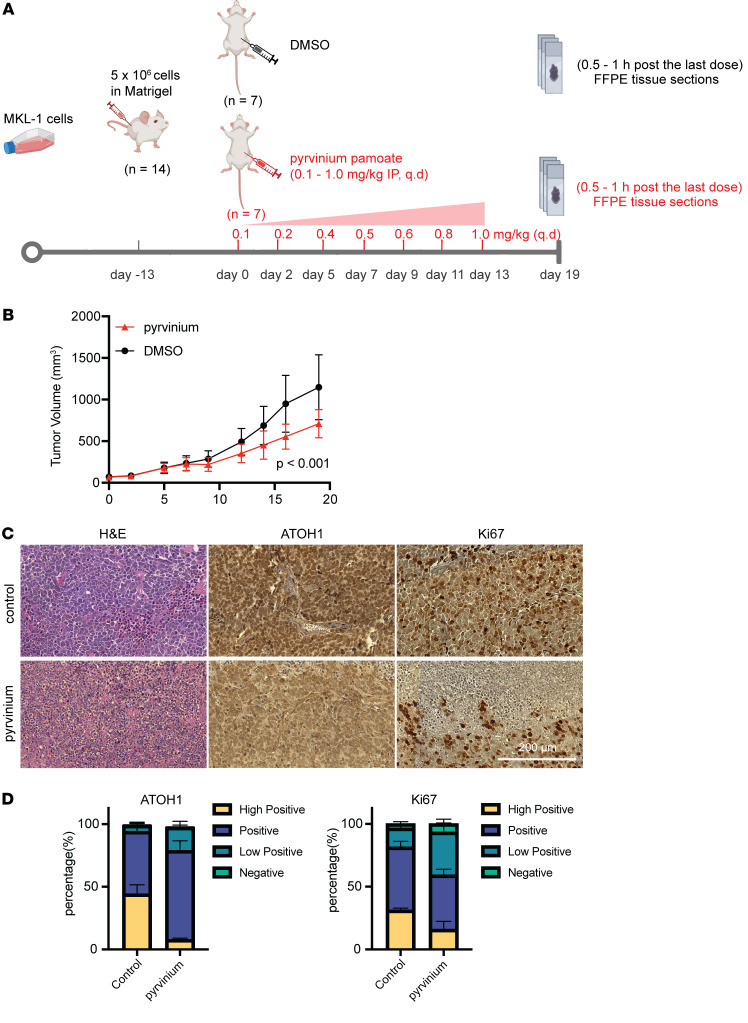
Antitumor activity of pyrvinium in an MKL-1 xenograft tumor model. (**A**) Experimental design of the in vivo study. (**B**) Tumor growth curve showing the mean tumor volume of vehicle control and pyrvinium-treated mice from day 0 to day 20 of treatment (mean ± SEM, *n* = 7). (**C**) H&E and IHC staining results on serial sectioning slides for each marker in the same lesion. Tumor tissues were collected 0.5 to 1 hour after the final 1 mg/kg dose of pyrvinium in in vivo Study number 2 (experimental design shown in Supplemental Figure 14C). (**D**) Percentage of MKL-1 xenograft tumor tissue expressing ATOH1 or Ki67 in vehicle control group and pyrvinium-treated group.

To assess the effect of pyrvinium on MCC cells in vivo, we performed a xenograft study with MKL-1 cells in NSG mice (Figure 7A). The administration of gradually increasing doses of pyrvinium, from 0.1 mg/kg to 1.0 mg/kg daily via intraperitoneal injection, was enough to cause tumor growth inhibition; the treated mice exhibited significantly slower tumor growth across time than the control mice (*n* = 7, *P* < 0.001) (Figure 7B and Supplemental Figure 13, A and B), with control mice exhibiting a slope of 1.34 (SE = 0.0823), while treated mice exhibited a slope of 0.937 (SE = 0.0861). 2 other independent studies, in which we administered higher doses of pyrvinium from 0.6 mg/kg to 1.0 mg/kg of pyrvinium daily (*n* = 4, *P* < 0.001, control slope: 2.61, treated slope: 1.43), or 1.0 mg/kg 3 times a week (*n* = 10, *P* < 0.001, control slope: 2.24, treated slope: 1.72) (Supplemental Figure 14, A–E, and Supplemental Tables 2–4), both demonstrated even larger reductions in tumor burden. Among these 3 independent studies, the gradual dose escalation strategy was optimally tolerated by NSG mice and resulted in minimal side effects. To test whether pyrvinium acts through the pathways identified in vitro, we performed H&E and IHC staining for ATOH1 and Ki67 on the xenograft tumors from the control and treatment groups (Figure 7C). We observed that pyrvinium-treated MCC tumors exhibited a modest decrease in ATOH1 expression, coupled with decreased levels of Ki67, thus demonstrating reduced proliferation in drug-treated tumors (Figure 7D). These in vivo results align with our in silico and in vitro findings.

## Discussion

In this study, we used an inducible cell line model to identify cellular pathways driving Merkel cell carcinoma. Leveraging genomic analyses and multiple databases, we shed light on the role of the Wnt signaling pathway and discovered that MCC is sensitive to the Wnt inhibitor pyrvinium pamoate. By studying the impact of pyrvinium on MCC cells, we discovered specific elements of canonical and noncanonical Wnt signaling that help maintain the neuroendocrine features of MCC. Pyrvinium also has antitumor effects through multiple mechanisms, including the activation of p53, downregulation of mitochondrial complex genes, and induction of endoplasmic reticulum stress. These insights contribute to our understanding of MCC development and offer potential avenues for targeted therapeutic strategies for this aggressive neuroendocrine malignancy.

In human skin, Wnt signaling enables intercellular communication between keratinocytes and fibroblasts to induce proliferation of dermal cells and regeneration of hair follicles (67) and Merkel cells (68). Previous research has demonstrated that, although β-catenin activity level is low in MCC tumors, canonical Wnt signaling can stimulate MCPyV infection in human dermal fibroblasts (17). It is known that many members of canonical and noncanonical Wnt signaling are expressed in the developing and mature nervous systems (69, 70). We found that aspects of both canonical and noncanonical Wnt signaling are altered in Merkel cell carcinoma. Using our IMR90 cell line model expressing MCPyV-ER, we discovered that canonical Wnt signaling genes, including *WNT3, TCF7*, and *TCF3*, are associated with neuroendocrine marker expression, whereas noncanonical Wnt genes, like *WNT5A*, *WNT5B*, and *WNT16* are negatively correlated with MCC markers, consistent with previous findings of low WNT5A expression in MCC cells (35). In particular, YAP and WWTR1 are 2 hippo pathway regulators that have growth-suppressive properties and are silenced in many NE cancers and MCCP (71, 72). It has been reported that YAP and WWTR1 are also mediators of noncanonical Wnt signaling (72). Consistent with this hypothesis, in IMR90-ER cells, we found that *WNT5B* expression is positively correlated with the expression of *YAP* and *WWTR1* and is negatively correlated with NE markers (Supplemental Figure 5B). We further observed that reducing TCF activity using siRNAs and increasing WNT5B through recombinant protein or an overexpressing vector in MCC cells exerts inhibitory effects on MCC marker genes and canonical Wnt activity. Overexpressing WNT5B over time in MCC cells inhibits cell growth. These results together suggest that the observed alterations in Wnt signaling play an important function in maintaining the neuroendocrine features of MCC. Since noncanonical Wnt signaling is known to induce terminal neuron differentiation, it is possible that Merkel cell carcinoma tumors must suppress noncanonical Wnts to remain in a proliferative progenitor-like cell state, while maintaining canonical Wnt (or TCF) activity at a level that supports proliferation. However, further experiments are needed to determine how noncanonical Wnt ligands are suppressed during MCC development.

Our IMR90 cell line model has several limitations, including the fact that IMR90s are derived from embryonic lung tissue and that we only profiled their transcriptome for 48 hours after early region expression, whereas MCPyV is a lifelong infection and MCC tumor development occurs over a timescale of years. To mitigate these issues, we confirmed the observed changes to the Wnt pathway in neonatal human dermal fibroblasts (nHDFs) and MCC tumors. Moreover, in both IMR90s and nHDFs, we observed that the response of the host transcriptome stabilizes by 48 hours after induction of MCPyV-ER, with little change observed between 48 to 96 hours (59). This suggests that our experimental design sufficiently captures the immediate response of the host cell to MCPyV ER. However, expression of MCPyV-ER over months or years could cause epigenetic alterations and other long-term changes that promote MCC development. We leave this question to future work.

Next, we used bioinformatic analysis of the LINCS L1000 dataset to identify pyrvinium pamoate, an FDA-approved anthelminthic drug and known inhibitor of canonical Wnt signaling, as a therapeutic candidate for MCC. Pyrvinium reduces the activity of canonical Wnt master regulators TCF3 and TCF7 and increases expression of noncanonical Wnt ligands WNT5A/B. Moreover, analysis of LINCS L1000 genetic perturbation data, samples from patients with MCC, and RNA-seq data from pyrvinium-treated MCC cell lines suggests that pyrvinium’s effect in MCC might involve a combined perturbation of multiple Wnt components (Supplemental Figure 9G). To further elucidate the relationship between pyrvinium, Wnt signaling, and MCC, future experiments could determine target genes of TCF3/7 using ChIP-seq or ATAC-seq and systematically measure the activity of key components of the canonical and noncanonical Wnt pathways — such as β-catenin, CK1α, WNT5A/B and their isoforms, and NFATs — upon T antigen expression or pyrvinium treatment.

We found that pyrvinium acts through both p53-dependent and -independent pathways to inhibit MCC growth. Because of the relatively low *TP53* mutation rate in MCC, particularly in MCCP, compared with other cancer types, the ability of pyrvinium to activate p53 response could prove beneficial (2, 73). Prior studies applying the ubiquitin ligase MDM2 inhibitors alone or with MDM4 inhibitors have led to p53 activation and cell apoptosis (9, 10, 65). Through transcriptomic profiling of pyrvinium-treated MCC cells, we observed significant activation of p53 signaling and validated it at protein levels. Pyrvinium exhibited similar or even higher efficacy for p53 activation and p53-mediated cell apoptosis than the MDM2 inhibitor Nutlin-3a in *TP53*^WT^ MCC cells. Pyrvinium is known as an activator of CK1α, a serine/threonine protein kinase, the activation of which promotes the phosphorylation and degradation of β-catenin by proteasomes (44). Prior work has shown that MCPyV ST can induce the overexpression of CK1α in MCC (9). Notably, other studies determined that CK1α could phosphorylate the N-terminal phosphorylation sites of p53, especially at the serine 20 site, which is believed to attenuate interaction of p53 with MDM2 and stabilize the binding of the coactivator p300, thereby activating p53 function (74, 75). CK1α activation could be one of the mechanisms by which pyrvinium activates p53, but further experiments are required to elucidate this.

Our analyses revealed that pyrvinium efficiently inhibited oxidative phosphorylation in MCC cells at an effective dose as low as 50 nM. Both transcriptomic and protein-level assessments indicated that pyrvinium suppressed the transcription of mitochondrial DNA. This observation aligns with a previous study that demonstrated a correlation between pyrvinium efficacy and the expression of mitochondrial-related genes in other cell lines (38). A potential mechanism for mitochondrial inhibition could be pyrvinium binding to and stabilizing mitochondrial G-quadruplexes, thus disrupting mitochondrial transcription (38). We also revealed that pyrvinium induced apoptosis by enhancing endoplasmic reticulum stress and abrogating the UPR signaling by targeting GRP78. In addition to these MOAs, we also observed that pyrvinium downregulates expression of EZH2 and survivin (BIRC5), 2 previously reported drug targets in MCC (21, 25). Potential cotreatment with pyrvinium and EZH2 inhibitors should be tested in MCC models.

In a xenograft model, we demonstrated that pyrvinium effectively suppressed MCC tumor growth in NSG mice and concurrently reduced MCC marker genes within the xenograft tumor tissue. There remains a need for further optimizing the administration method — through either IP injection or oral delivery systems — since some of the mice in our higher-dose studies did not tolerate IP injection at 0.6–1 mg/kg dosage. Encouragingly, pyrvinium pamoate was reported safe with oral dosing with the best effects noted at 35 mg/kg daily in mice (38). Moreover, pyrvinium pamoate has received approval for a Phase 1 clinical trial aimed at treating pancreatic ductal adenocarcinoma (PDAC) (NCT05055323), showing that a safe treatment protocol is possible for human patients (76). Our in vivo data, combined with other published reports, highlights pyrvinium as a candidate for anticancer therapy in Merkel cell carcinoma.

Through a combination of genomic studies, bioinformatics, and in vitro and in vivo work, our research has shown that the Wnt signaling pathway plays a functional role in maintaining the neuroendocrine features of Merkel cell carcinoma. Furthermore, we have demonstrated the potential of pyrvinium pamoate as an antitumor agent that targets multiple vulnerabilities of MCC. Further studies are needed to comprehensively characterize the role of Wnt signaling on cancer hallmarks and to optimize treatment protocols for the development of pyrvinium pamoate as a clinically useful drug for Merkel cell carcinoma.

## Methods

### Sex as a biological variable.

Sex was not considered as a biological variable. Experiments were conducted on female and male mice in separate studies, and similar results were observed in both sexes.

### Cell culture and chemicals.

Characteristics of IMR90 cells, nHDF cells, and Merkel cell carcinoma cell lines WaGa, MKL-1, MS-1, and MKL-2 have been previously reported (17, 20, 59). MCC cell lines WaGa, MKL-1, MS-1, and MKL-2, as well as fibroblast cell line IMR90 have been previously described (77). Fibroblast cell line nHDF was obtained from ATCC (PCS-201-010). All the cell lines were cultured at 37°C under 5% CO_2_. IMR90 cells were cultured in DMEM complete medium (Corning, cat 10-013-CV), containing 15% FBS, 10 U/mL penicillin, and 10 mg/mL streptomycin (GIBCO, cat 35050061). nHDF cells were cultured in DMEM complete medium, containing 10% FBS, 1% GlutaMAX, 10 U/mL penicillin, and 10 mg/mL streptomycin. WaGa, MKL-1, MS-1 and MKL-2 cells were cultured in RPMI-1640 medium (Corning, cat 10-040-CV), containing 10% FBS, 1% GlutaMAX, 10 U/mL penicillin, and 10 mg/mL streptomycin; See Supplemental Table 5 for chemicals used in this study.

### Cell transfection.

Doxcycline-inducible IMR90 and nHDF lines expressing MCPyV-ER and GFP (or vector control) were generated as previously described (59). The inducible plasmids containing MCPyV-ER and GFP were generated by cloning PCR products of the MCPyV early region DNA sequence, isolated from MCC tumor sample MCCL21 (NCBI accession number: KC426955), and EGFP sequence into the pLIX_402 donor vector using the Gateway system. WaGa cell lines expressing 7xTcf-eGFP (WaGa TopGFP) and hWNT5B (WaGa WNT5B OE) were generated with Mirus Bio TransITLenti Transfection Reagent (Thermo Fisher Scientific, cat MIR6604), according to the manufacturer’s instruction. 7TGP reporter plasmid was a gift from Roel Nusse (Addgene plasmid 24305), and the inducible WNT5B overexpression plasmid was generated by cloning the hWNT5B[NM_030775.2] protein coding sequence into the Gateway pLIX_403 donor vector. pLIX_403 donor vector plasmid was a gift from David Root (Addgene plasmid 41395). Lentiviral packaging plasmid psPAX2 and envelope plasmid pMD2.G were gifts from Didier Trono (Addgene plasmids 12260 and 12259). The siRNAs for TCF3, TCF7 and negative control (IDT, cat 51-01-14-03) were resuspended in Nuclease-Free Duplex Buffer (IDT, cat 11-01-03-01) to achieve a stock concentration of 100 μM. Transfection was performed using the Neon Transfection system (Thermo Fisher Scientific, cat MPK5000, and MPK100). For each transfection, 2 μL of siRNA was added to 100 μL of 1 million WaGa cells in DPBS. A single electrical pulse of 1800 V for 20 ms was applied to introduce the siRNA into the cells. The electroporated cells were then transferred into 2 mL of fresh complete culture medium, resulting in a final siRNA concentration of 10 nM. After 48 hours of culture, the cells were collected for assays. The full sequences of all plasmid constructs, siRNAs, and primers are provided in the Supplemental Materials.

### Western blot.

Cells were seeded in 25 cm^2^ cell culture flasks and were treated with pyrvinium for 24 hours. Then, cells were washed with cold PBS and lysed with RIPA buffer (Thermo Fisher Scientific, cat 89901) supplemented with EDTA-free protease inhibitor cocktail (Roche, cat 04693132001). After centrifugation for 20 minutes at 32,310*g* at 4°C, supernatant was collected. Protein concentrations were determined using the Pierce BCA assay kit (Thermo Fisher Scientific, cat 23227). Protein samples were denatured with 4 × Laemmli Sample buffer with 10% β-mercaptoethanol (Sigma-Aldrich, cat M6250) followed by 10 minutes boiling at 95°C. Then, samples were loaded and run on SDS-PAGE for 80 minutes at 120 V. Proteins were transferred to nitrocellulose membranes at 200 mA for 90 minutes. Blocking and incubation of primary and secondary antibody were performed under manufacturer-recommended conditions. The membranes were visualized by Li-Cor Odyssey FC imager. See Supplemental Table 6 for antibodies used in this study. Every blot was repeated at least 2 times, and quantification and statistics are provided in Supplemental Spreadsheet 8.

### Mitochondrial oxygen consumption rate (OCR) analysis.

The assay plates and cartridges were treated with 1mL of Seahorse XF96 calibrant overnight in a 37°C non-CO_2_ incubator, ensuring that each well was fully immersed in liquid (Seahorse Bioscience, cat 103680-100). WaGa cells were washed by DPBS once and resuspended in fresh culture media to 3 × 10^6^ cells/mL. The cell suspension was aliquoted into different 15 mL tubes and the pyrvinium was added to final concentrations of 10, 30, 50, 100, 200, 300, 500 nM, and 1 μM. A multichannel pipette was used to transfer 50 μL of the cell suspension (1.5 × 10^5^ cells) evenly to each well of the PDL plates (Seahorse Bioscience, cat 103798-100). The cells were centrifuged at 200*g* (zero braking) for 1 minute and plates were transferred to a 37°C incubator. After 24 hours of treatment, the PDL plates were gently tapped on stacks of tissue paper to get rid of the drug containing culture media. Then, 180 μL of warm assay media (Seahorse Bioscience, cat 103681-100) was slowly and gently added along the side of each well after washing the cell with assay media once. The plates were placed in the incubator for 30 minutes and cells were observed under the microscope to check that cells were not detached and 95% of the well bottom area was covered by WaGa cells. Maximal respiratory capacity (FCCP-OCR) was measured using the Seahorse Bioscience XF-96 Extracellular Flux Analyzer (Seahorse Bioscience). The protocol is optimized for the given condition; basal OCR was measured 3 times followed by injection of 20 μL of 5 μM FCCP (Sigma-Aldrich, cat C2920) in port A to reach a final concentration of 0.5 μM. The stepwise settings used to measure the FCCP-OCR in the WaGa cells are shown in Supplemental Table 7.

### Quantitative real-time PCR.

Total cellular RNA was extracted using TRIzol reagent (Invitrogen cat 15596026) according to the manufacturer’s instructions. Then, the RNA samples were reverse transcribed into cDNA using the SuperScript IV RT-PCR kit (Thermo Fisher Scientific, cat 12594100). Real-time PCR was then performed using the Applied Biosystems StepOne system with SYBR Green RT-PCR Master Mixes (Thermo Fisher Scientific, cat A25742). The PrimePCR SYBR green assay primers (see Supplemental Table 8) were used to amplify gene of interests and housekeeping gene. The data were acquired as a threshold cycle (Ct) value. The ΔCt values were determined by subtracting the average internal housekeeping gene Ct value from the average target gene Ct value. Since the amplification efficiency of the target genes and internal control gene was equal, the relative gene expression was calculated using the 2^–ΔΔCt^ method. Each measurement was performed in triplicate and repeated 3 times.

### Xenograft efficacy study.

5 × 10^6^ MKL-1 cells with 50% Matrigel were implanted in the right flank of 8-week-old NSG mice (The Jackson Laboratory, Strain 005557) subcutaneously. Tumors were allowed to grow to an average size range 50–100 mm^3^ until randomized into 2 groups per group. Mice were treated with vehicle control (10% DMSO + 90% of 20% HP-β-CD) or pyrvinium pamoate administered intraperitoneally once daily. We used both female and male mice in 3 independent studies. In Study number 1, male mice were treated with 1 mg/kg pyrvinium 3 times (MWF) a week (*n* = 10). In Study number 2, female mice were treated at 0.6 mg/kg for 14 days, followed by 7 days at 1 mg/kg dose (*n* = 4). In Study number 3, male mice were treated with increasing doses, starting at 0.1 mg/kg on day 1, then escalating to 0.2 mg/kg on day 3, 0.4 mg/kg on day 6, 0.6 mg/kg on day 8, 0.8 mg/kg on day 11, and finally 1.0 mg/kg from day 13 until the study ended on day 19 (*n* = 7). All control and treated mice received subcutaneous injections of saline concurrently with each dose. For all 3 studies, tumor volumes and body weights were measured 3 times a week. Tumor samples were collected from mice reaching endpoint (tumor volume exceeding 2,000 mm^3^) or on study termination on day 20. A third of each tumor sample was fixed using 10% neutral formalin and kept at room temperature for 24 hours. After 24 hours, samples were then transferred and preserved in 70% ethanol and proceeded to paraffin embedding for IHC. The rest of the tissue was snap frozen with liquid nitrogen and stored at –80°C for further analysis. Statistical analysis was performed using a linear mixed effects model to account for the correlation in the tumor volume measurements across time within a mouse. Tumor volume was transformed and normalized by using the square root. The model incorporates the effects of time, treatment, and their interaction; the interaction tested whether the tumor volume growth rate across time (slope) differed in the pyrvinium-treated versus control mice. A *P* value under 0.05 was considered statistically significant.

### RNA-seq.

IMR90 cells were transduced with doxycycline-inducible lentiviral vectors containing MCPyV-ER (with truncated large T antigen) or GFP sequence (as a control). The cells were treated with doxycycline for 48 hours and harvested at 0, 4, 8, 12, 16, 20, 24, 32, 40, and 48 hours after dox treatment, triplicated for each time point. RNA was purified using the RNeasy Plus Mini Kit (QIAGEN, cat 74034) and mRNA was isolated with NEBNext Poly(A) mRNA Magnetic Isolation Module (New England BioLabs, cat E7490). Sequencing libraries were prepared with the NEBNext mRNA library Prep Master Mix Set for Illumina (New England BioLabs, cat E6110), passed Qubit bioanalyzer and qPCR QC analyses, and sequenced on the Illumina HiSeq 2000 platform.

WaGa and MKL1 cells were treated with DMSO or 1 μM pyrvinium for 6 hours and 24 hours in triplicate for each condition. nHDF-ER and nHDF-Ctrl cells were treated with doxycycline for 0, 48, and 96 hours, also in triplicate for each time point. Total RNA was isolated from the cells using the RNeasy mini kit (QIAGEN, cat 74104). The isolated RNA was subjected to quality control using an Agilent 2100 bioanalyzer, and all samples passed the quality check. The RNA samples were then subjected to library preparation and sequencing on the Illumina NovaSeq 6000 platform.

### WGCNA.

Weighted gene coexpression network analysis (WGCNA) was performed using the WGCNA R package (78). A signed network was constructed on the 10,513 genes differentially expressed between the early region 48 hour and GFP 48 hour samples (with *P*_adj_ ≤ 0.05 and no threshold on fold change) across all time points. The soft thresholding power was set to 16 after comparing with scale-free topology. To identify modules of highly correlated genes, we applied a minimum module size of 30 and a height cutoff of 0.25 to the hierarchical clustering dendrogram. The algorithm assigned the 10,513 genes to 14 signed modules. The eigengene of each module was then projected onto individual samples, and the mean of the triplicates for each time point was used for plotting. GO enrichment analysis was performed on each module to identify biological pathways enriched among the genes within the module.

We then calculated the sum of the Topological Overlap Matrix (TOM) for all edges that connect to each gene and sorted genes based on this score. The 40 genes that had the highest summed edge weight in each module were selected as the hub genes for that module. Next, we further filtered the network by selecting the leading edges (the top quintile) ranked by TOM edge weight. The R package networkD3 was used to generate the force directed network on the filtered top hub genes.

### Regulatory network analysis.

We used PANDA (54), a method that integrates information from gene expression, protein-protein interaction, and transcription factor binding motif data, to generate aggregate gene regulatory networks for IMR90-ER (*n* = 29) and IMR90-GFP (*n* = 27) samples. Then, LIONESS (55) was applied to extract single-sample networks. To avoid issues caused by negative edge weights, we transformed the network edges using the following equation:
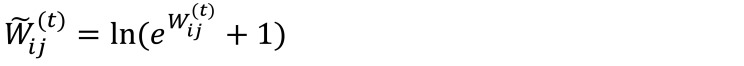


where the edge weight is calculated by PANDA and LIONESS between TF(*i*) and gene(*j*) in a single-sample network (*t*).

Next, samples for each cell line were divided into 5 time periods (t1 = [0h, 4h], t2 = [8h, 12h], t3 = [16h, 20h], t4 = [24h, 32h], t5 = [40h, 48h]) and an averaged network was calculated for all individual LIONESS networks from each period. ALPACA, a method for detecting significant changes in the community structure of weighted bipartite transcriptional networks, was then applied to compare the community structure of the averaged early region and GFP networks at different time periods (56). We kept and reported differential communities that included more than 30 nodes. For each detected differential community, we performed GO enrichment analysis and reported the GO terms with *P*_adj_ < 0.05.

### Statistics.

R (version 4.2.2) and GraphPad Prism (version 9.0.2) were used in all statistical tests for computational and in vitro analyses. Specific statistical methods and details are described in figure legends and include Student’s *t* test, Fisher’s exact test, Kruskal-Wallis *H* Test followed by Dunnett’s post hoc test, and ANOVA followed by Dunnett’s multiple comparison test. Stata/SE (version 15.1) was utilized for the mouse xenograft study, with a comprehensive description of the model outlined in the xenograft efficacy study methods section. A *P* value less than 0.05 was considered significant.

### Study approval.

This study was approved by the IACUC for the care and use of laboratory animals at the University of Arizona (protocol 2021-0772).

### Data availability.

The data generated in this study are publicly available in Gene Expression Omnibus (GEO) under accession numbers GSE130639, GSE229701 and GSE278335. Previously published data analyzed in this study were obtained from Gene Expression Omnibus (GEO) under accession numbers GSE39612 and GSE70138. The protein-protein interaction data used for regulatory network analysis were obtained from the STRING database (file: “9606.protein.links.v11.5.txt.gz”). The TF binding motif data for regulatory network analysis was obtained from the website https://sites.google.com/a/channing.harvard.edu/kimberlyglass/tools/resources using the link for the Human Motif Scan (Homo sapiens; hg38). All code and processed data are available on GitHub (https://github.com/JiawenYang16/pyrvinium_in_MCC; branch: main; commit ID: 4c0026689d62bfef875993b7bc6f193a60600e2d). Values for all data points in graphs are reported in the Supporting Data Values file.

## Author contributions

JY conceptualized and designed the study, developed methods, conducted experiments, analyzed data, drafted, and revised the manuscript. JTL designed the study, developed methods, and analyzed data. PVSR developed methods, conducted experiments, and analyzed data. MGC and GDPM conducted experiments. QP and HK assisted with methods development. CC assisted on statistics and data analysis. RGS provided resources and facilities for the research and assisted in study design. DJR assisted on statistics and revised the manuscript. PCG provided guidance on xenograft model development and revised the manuscript. JAD provided resources and facilities for the research and revised the manuscript for intellectual content. MP conceptualized and supervised the study and revised the manuscript for intellectual content.

## Supplementary Material

Supplemental data

Supplemental data set 1

Supplemental data set 2

Supplemental data set 3

Supplemental data set 4

Supplemental data set 5

Supplemental data set 6

Supplemental data set 7

Supplemental data set 8

Unedited blot and gel images

Supporting data values

## Version 1 02/11/2025

In-Press Preview

## Version 2 04/01/2025

Electronic publication
