# Supplementary material for: Integrative analysis reveals therapeutic potential of pyrvinium pamoate in Merkel cell carcinoma
Source: J Clin Invest. 2025 Feb 11;135(7):e177724. doi: 10.1172/JCI177724 (PMC11957690; doi:10.1172/JCI177724)

Full unedited blots for Figure 5D

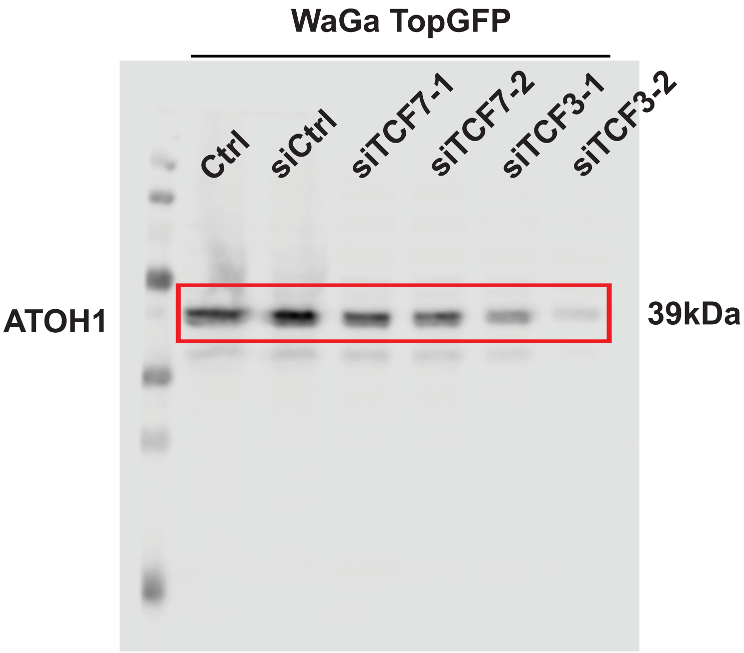

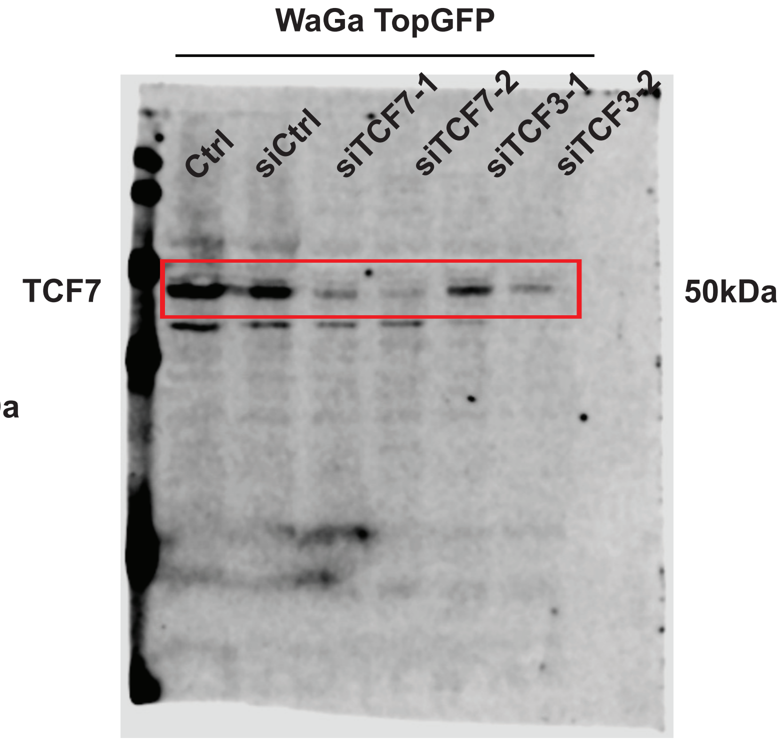

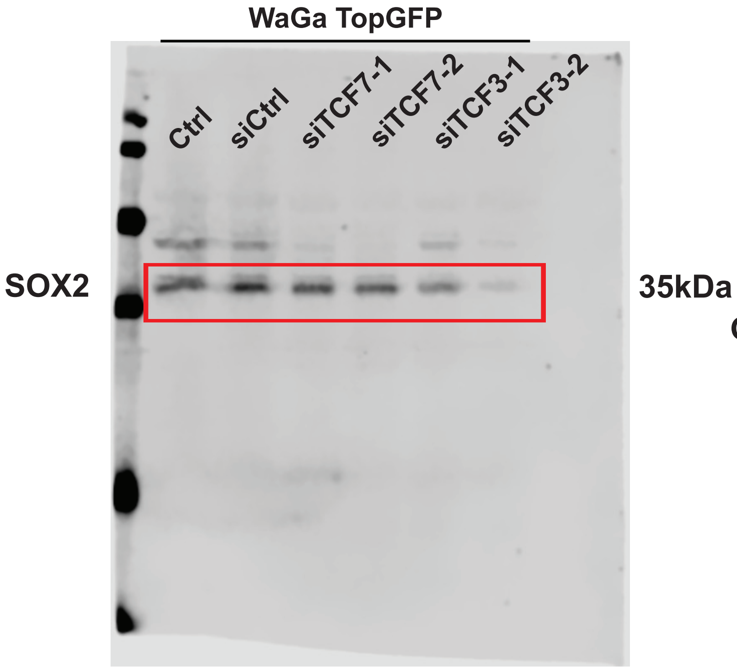

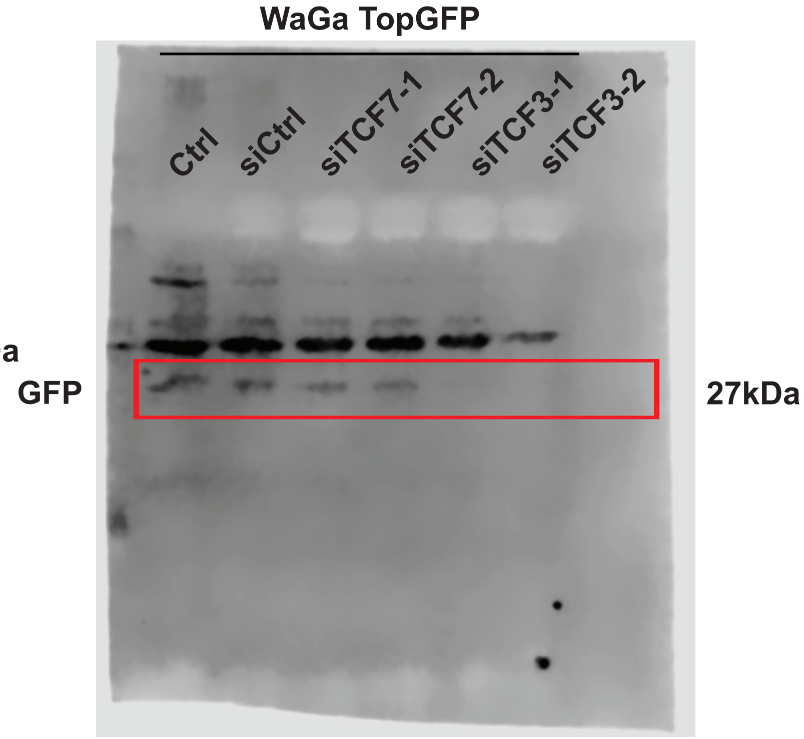

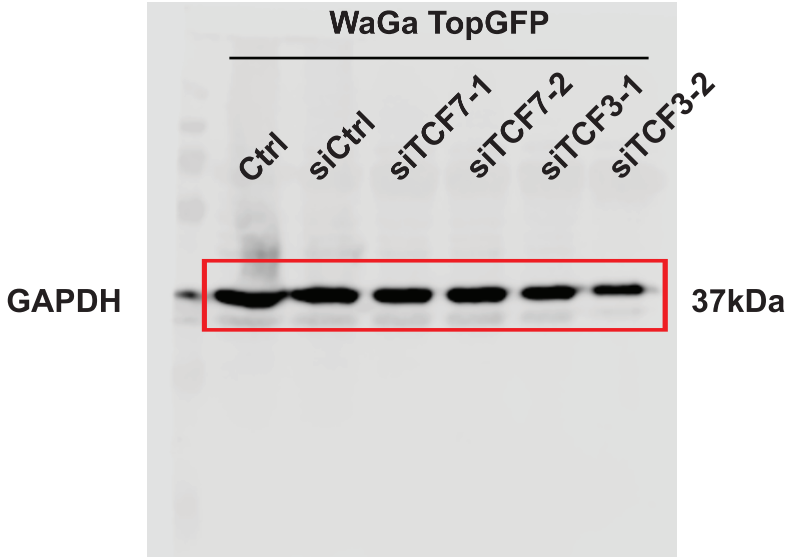

Full unedited blots for Figure 5E

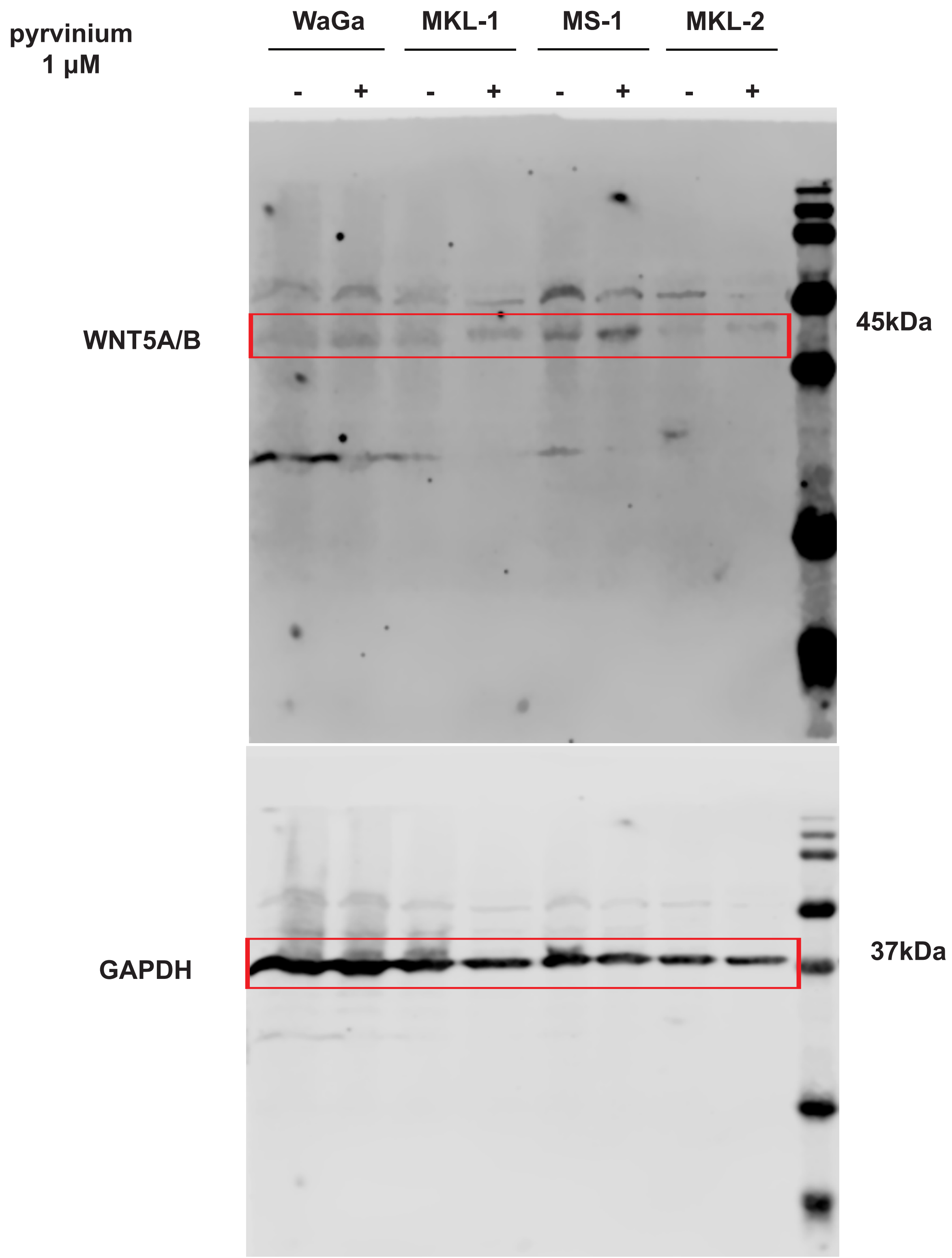

Full unedited blots for Figure 5G

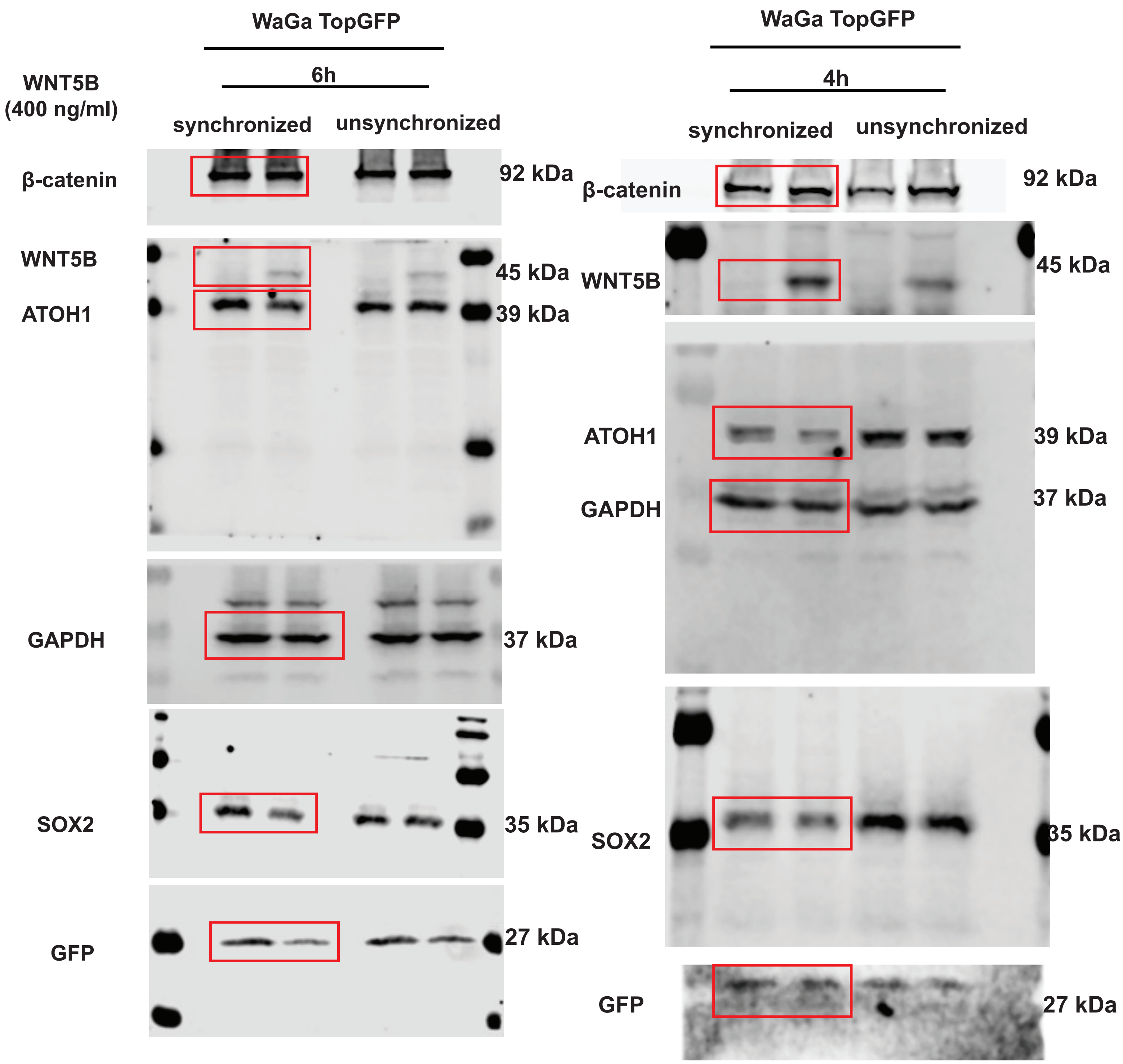

Full unedited blots for Figure 5H

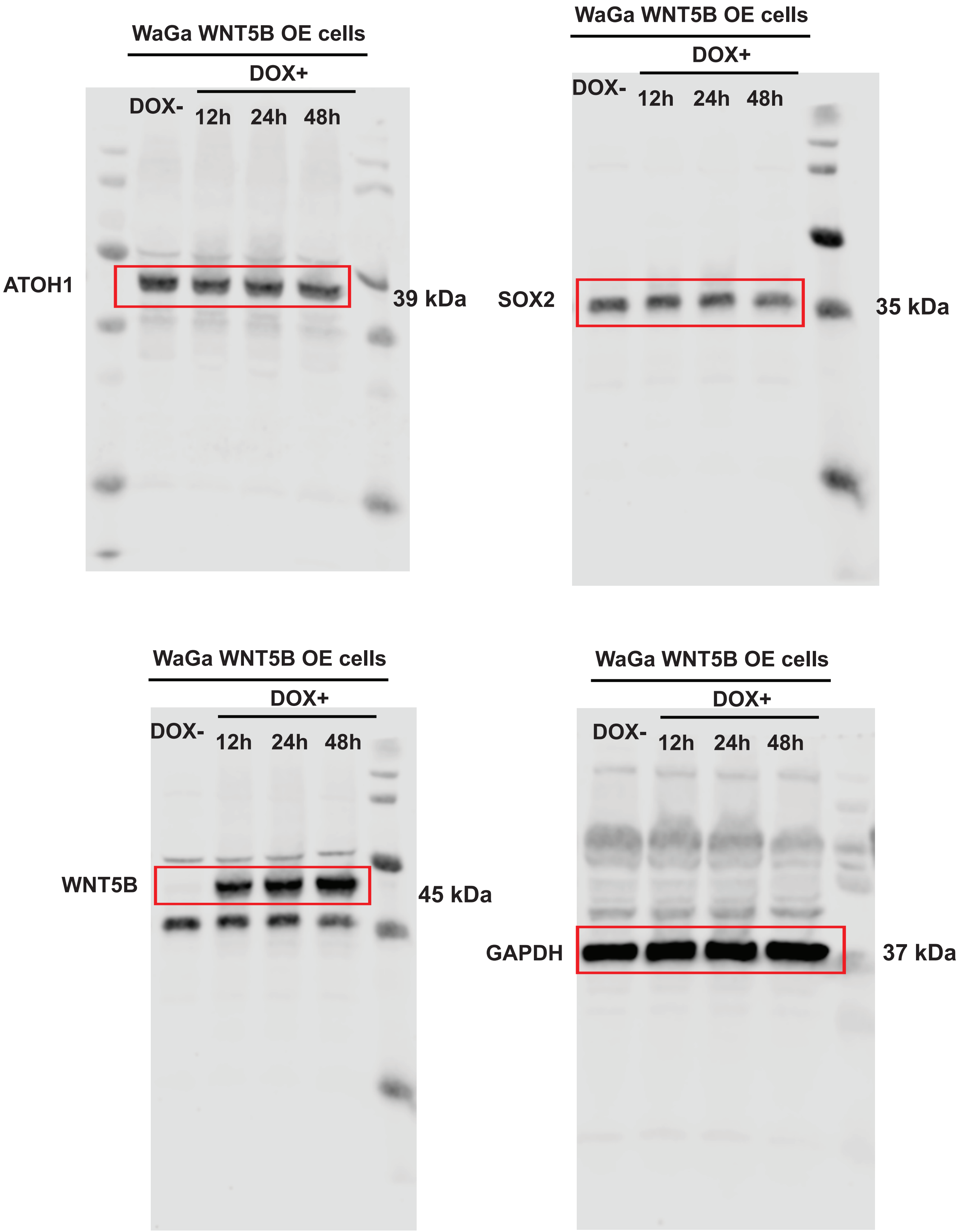

Full unedited blots for Figure 6B

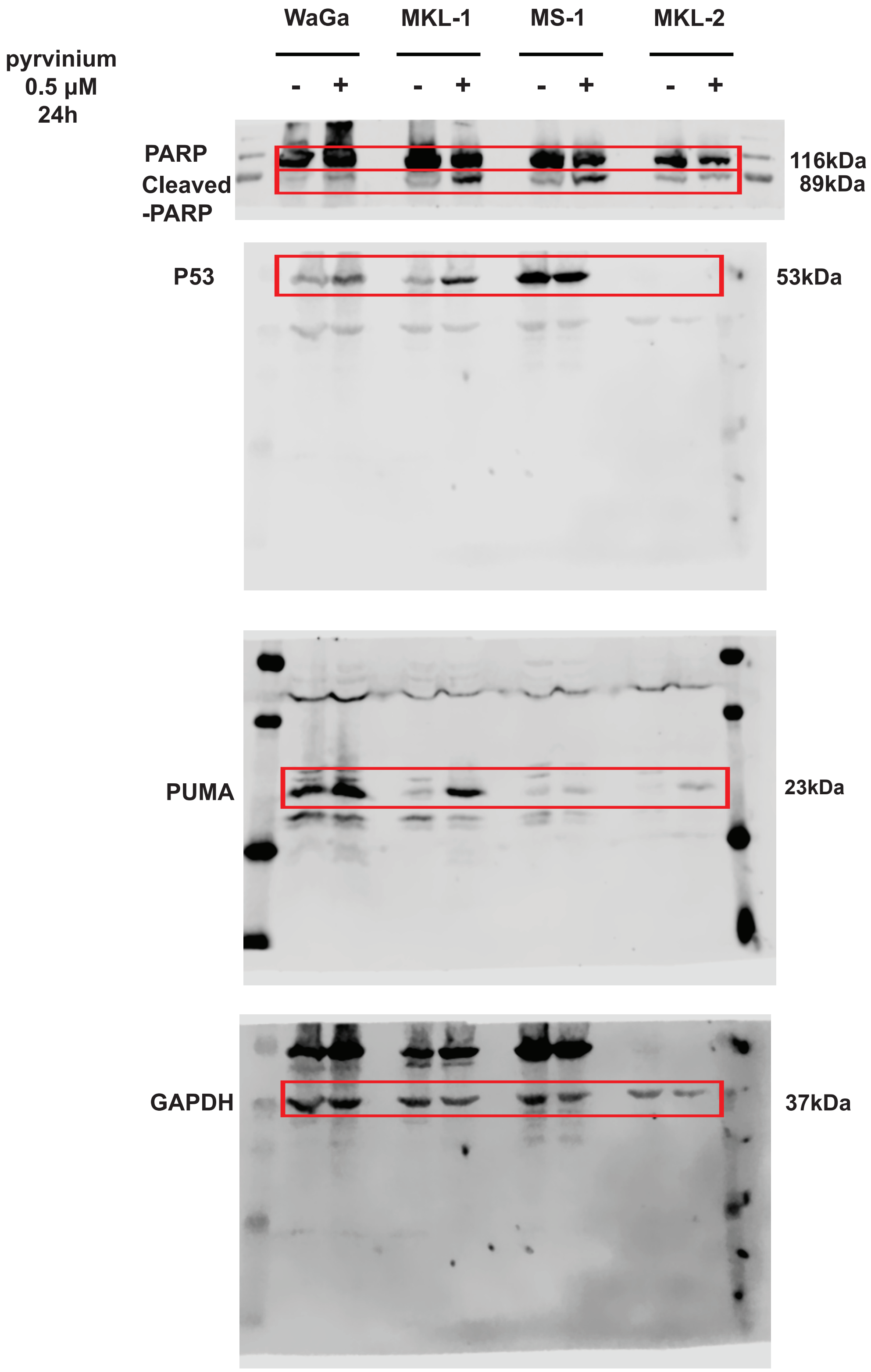

Full unedited blots for Figure 6C

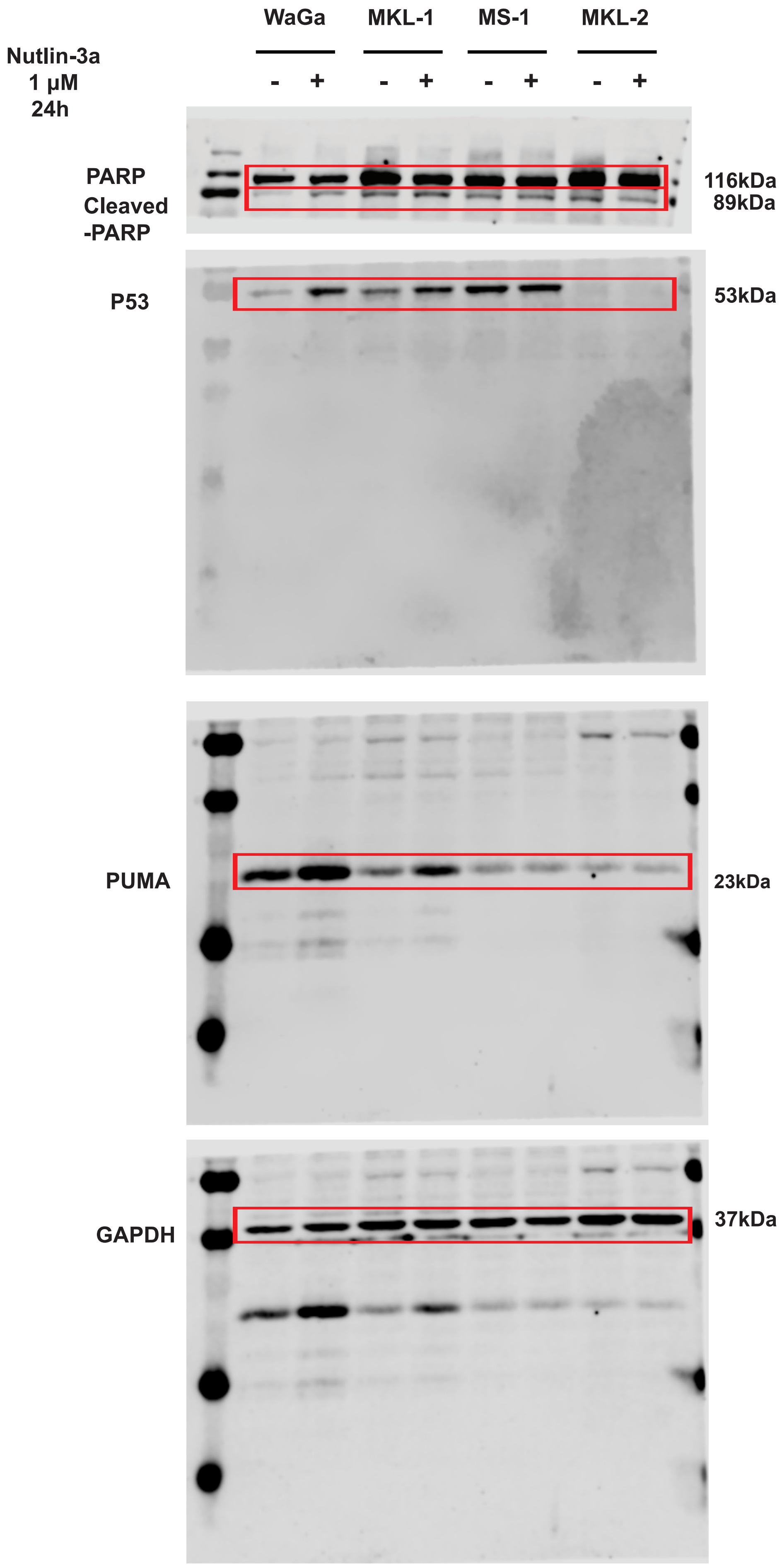

Full unedited blots for Figure 6G

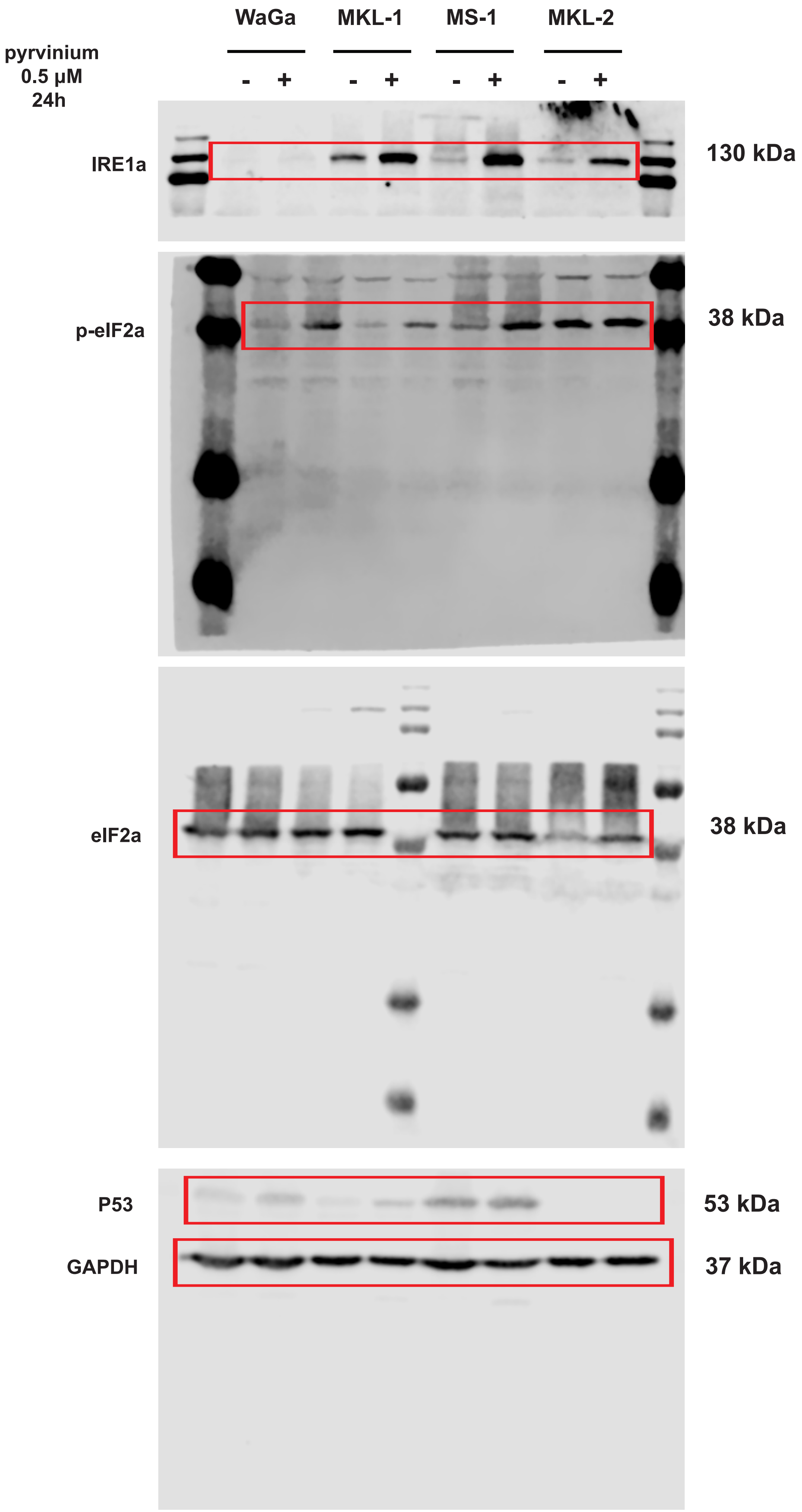

Full unedited blots for Figure 6I

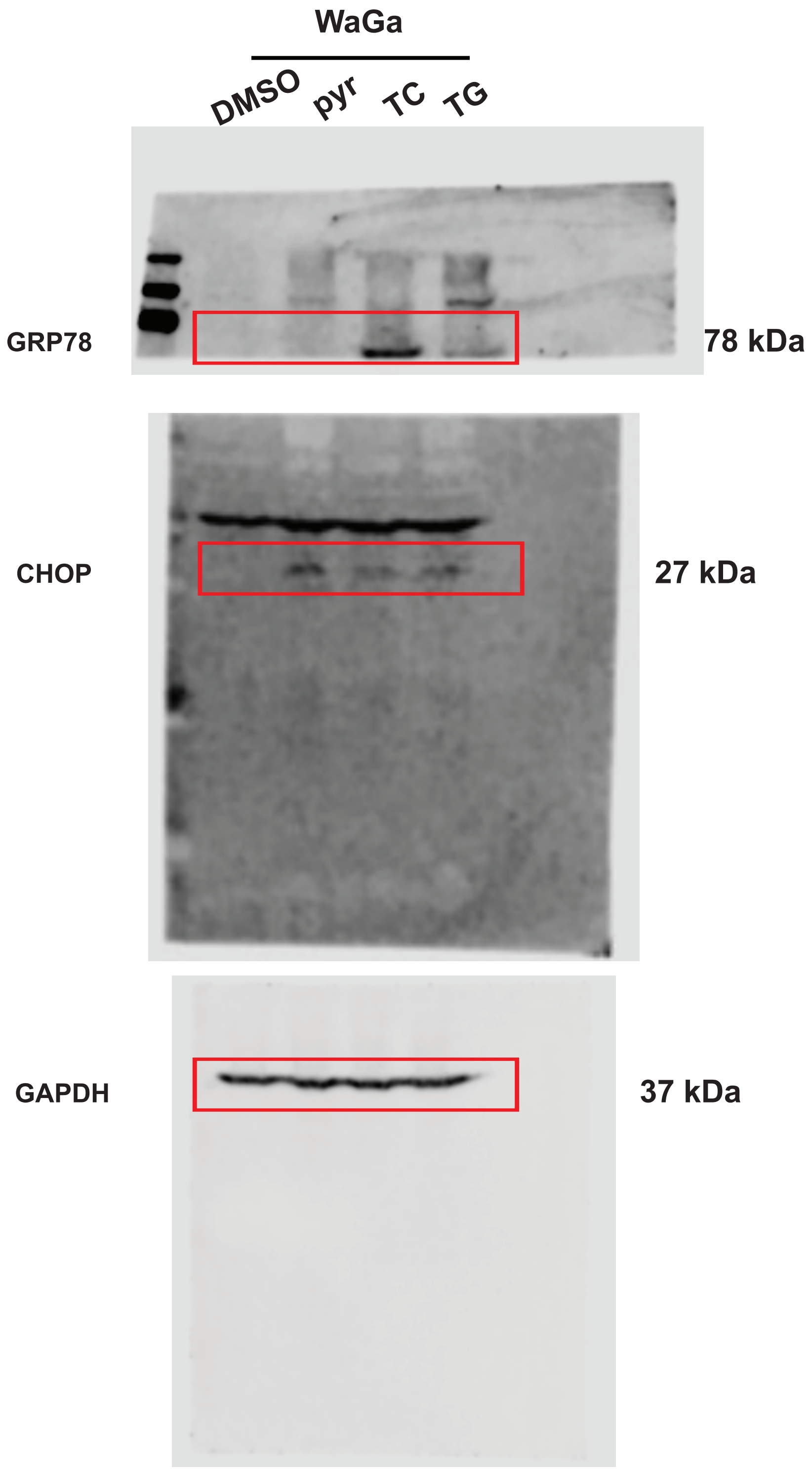

Full unedited blots for Supplemental Figure 6C

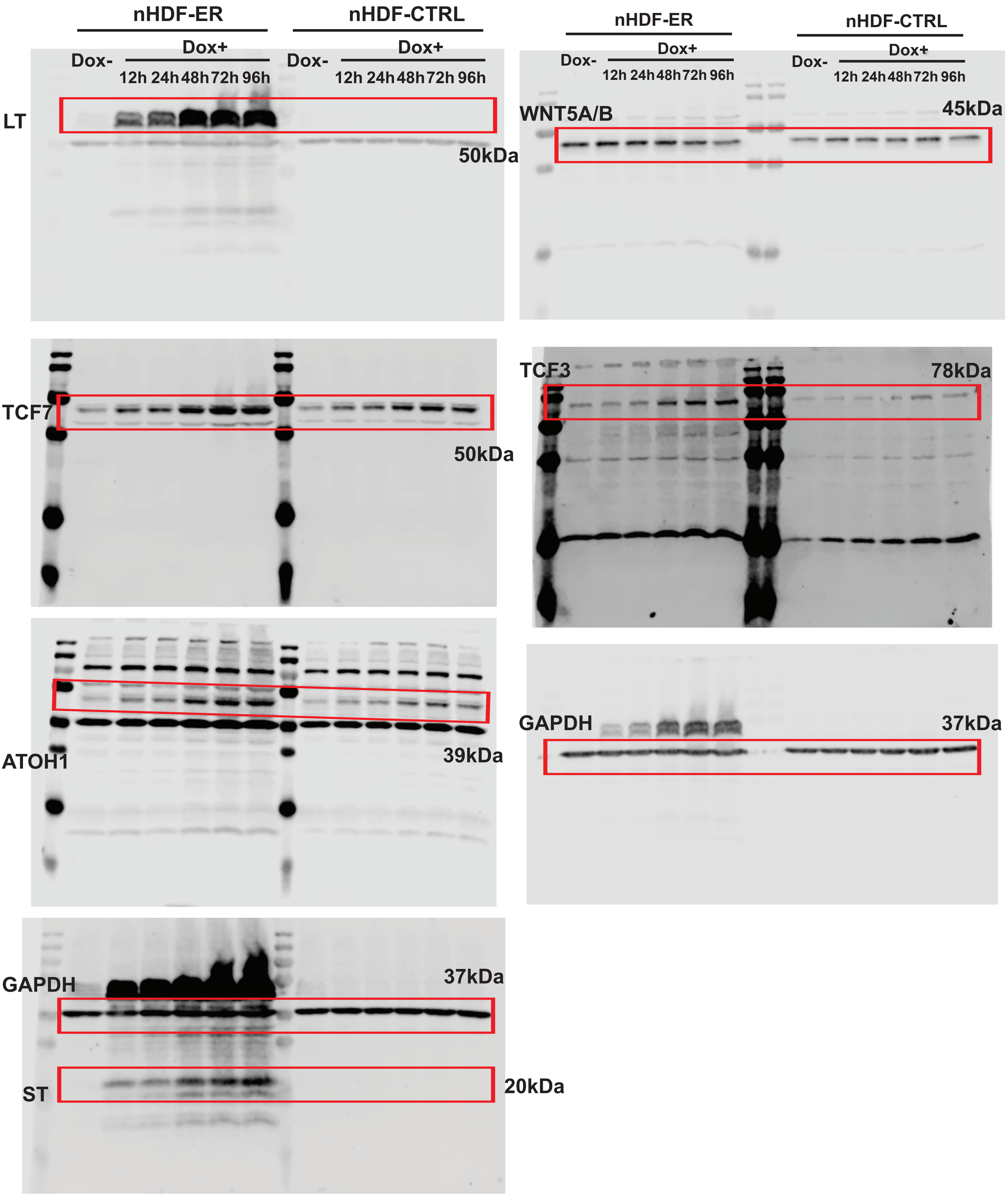

Full unedited blots for Supplemental Figure 9C

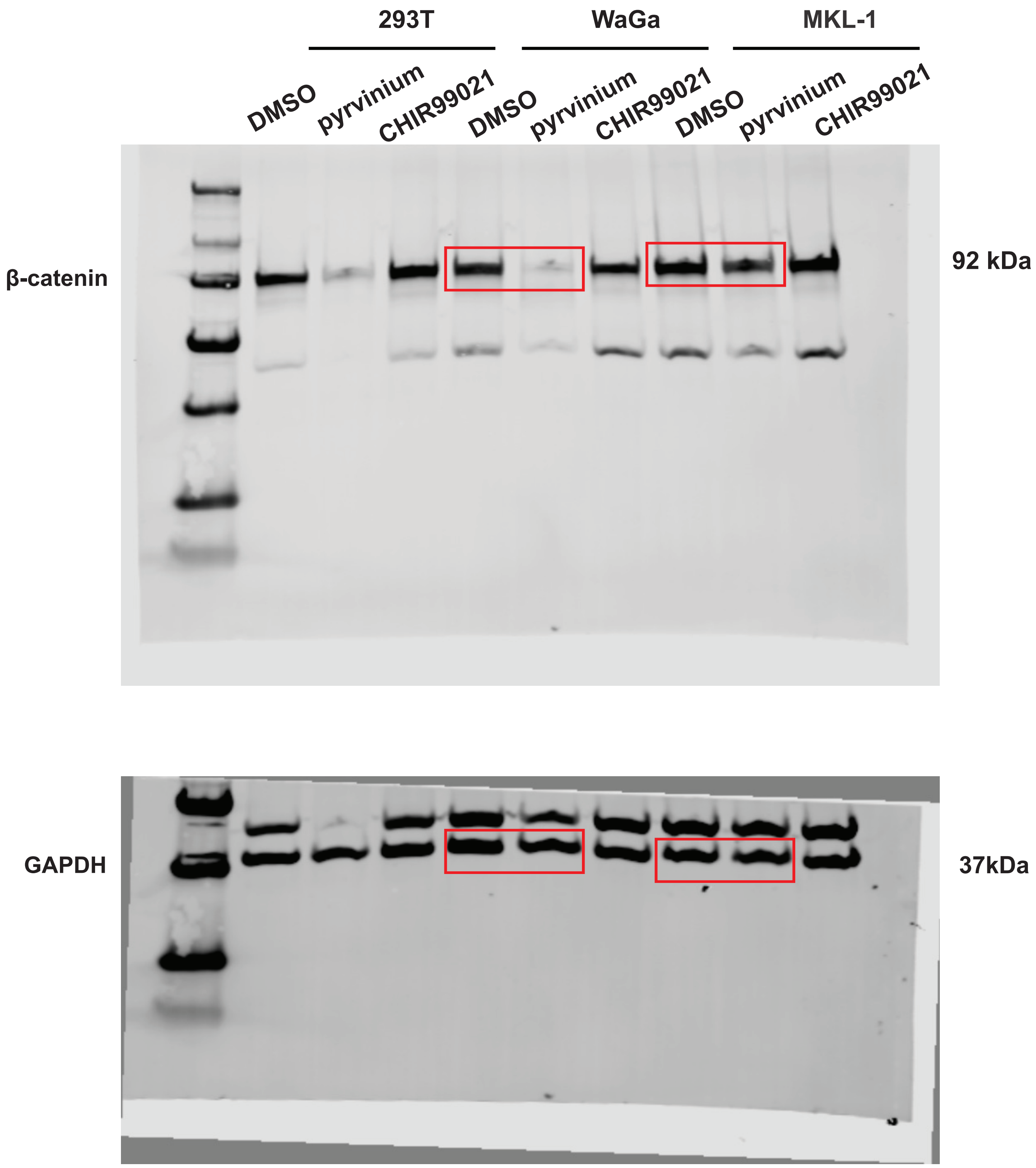

Full unedited blots for Supplemental Figure 9F

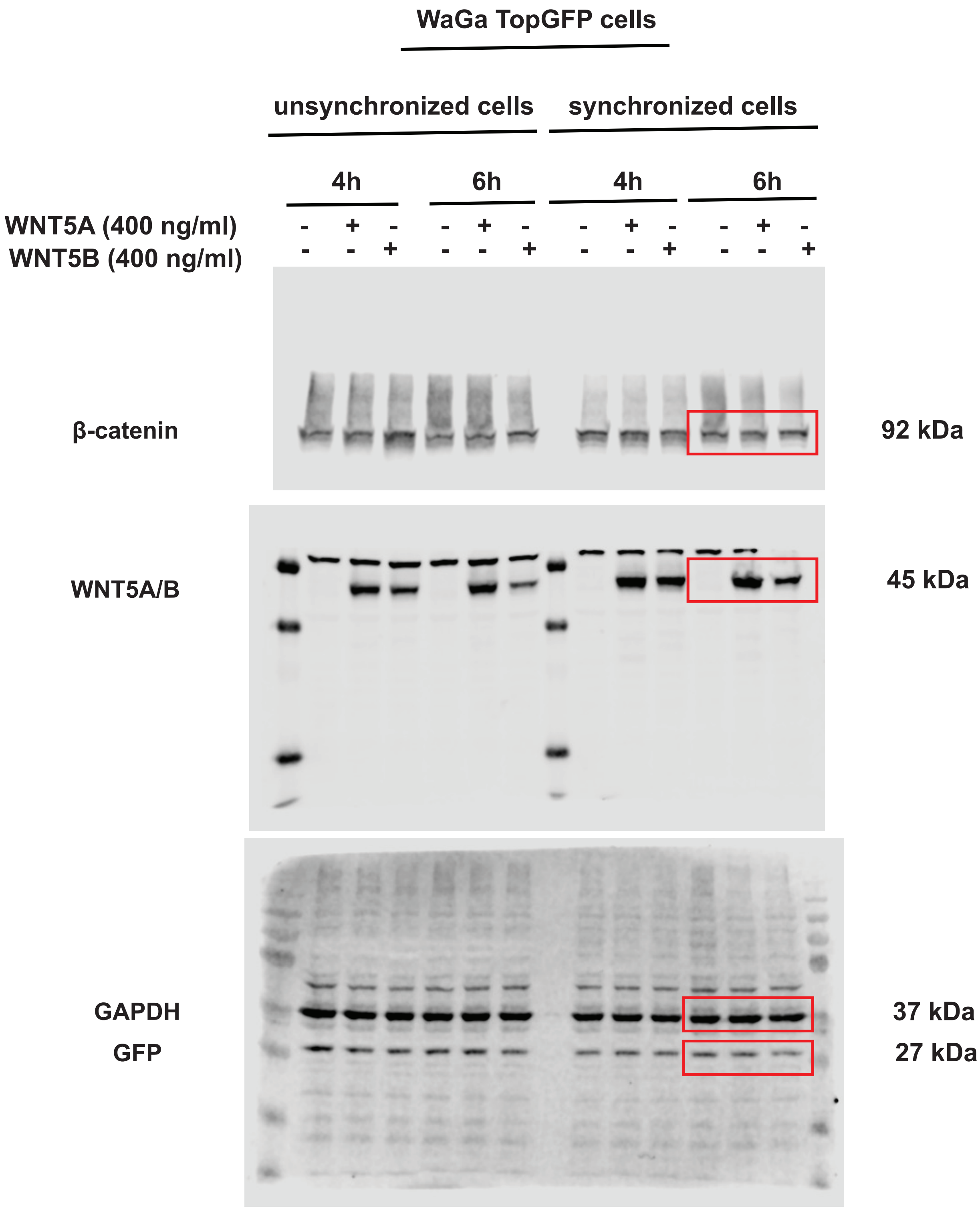

Full unedited blots for Supplemental Figure 12B

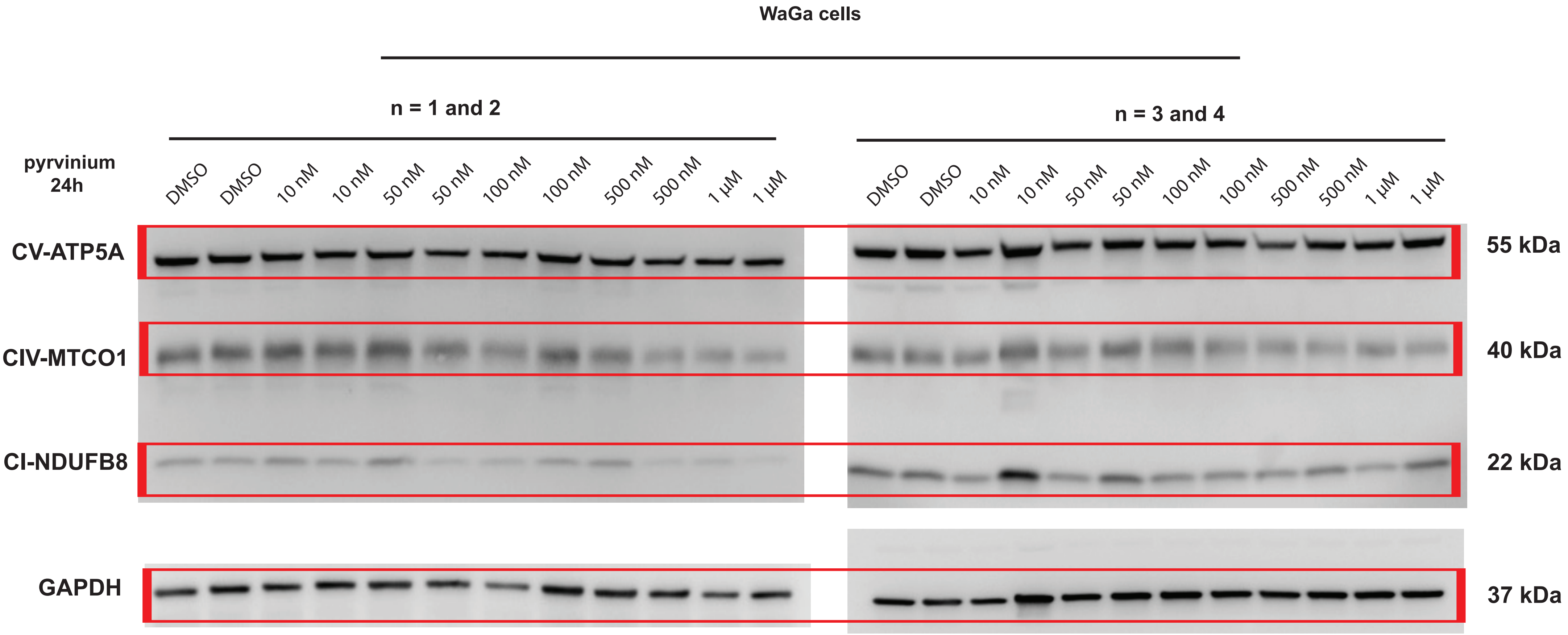

Full unedited blots for Supplemental Figure 12C

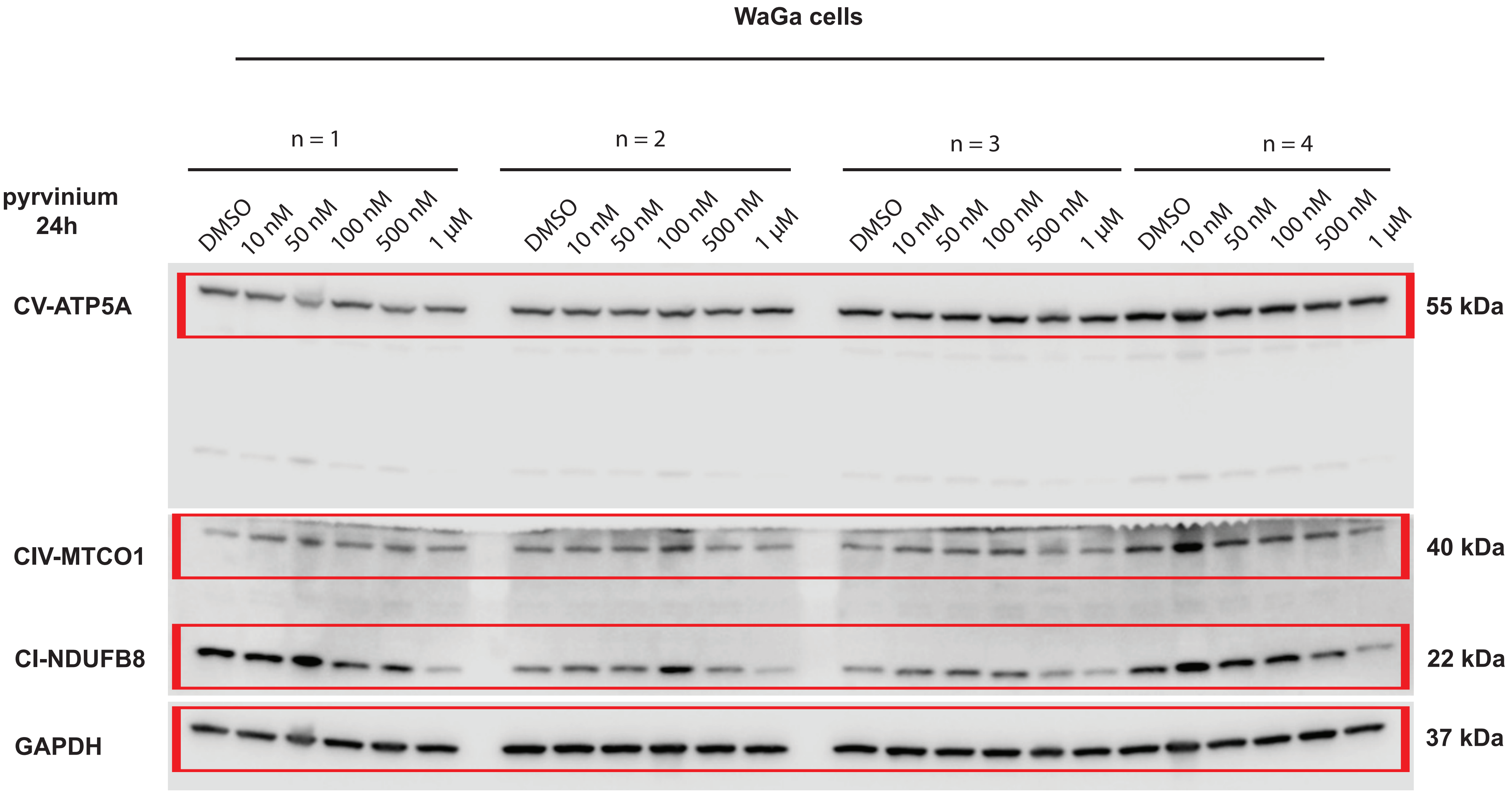

Full unedited blots for Supplemental Figure 12F

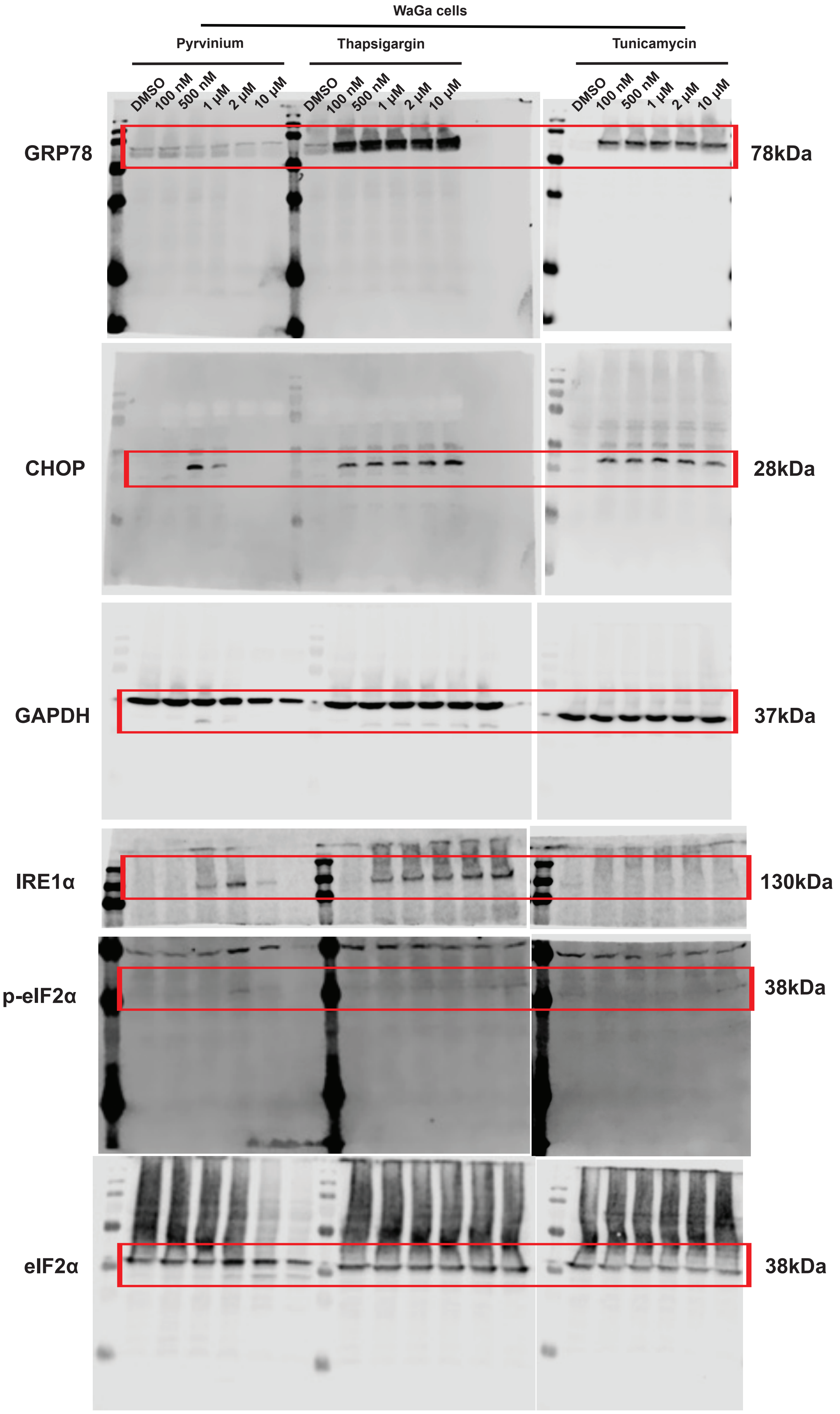

Supplement: Unedited blot and gel images [file jci-135-177724-s209.pdf]
